# Single-cell atlas of keratoconus corneas revealed aberrant transcriptional signatures and implicated mechanical stretch as a trigger for keratoconus pathogenesis

**DOI:** 10.1038/s41421-022-00397-z

**Published:** 2022-07-12

**Authors:** Shengqian Dou, Qun Wang, Bin Zhang, Chao Wei, Huijin Wang, Ting Liu, Haoyun Duan, Hui Jiang, Mingna Liu, Xiaolin Qi, Qingjun Zhou, Lixin Xie, Weiyun Shi, Hua Gao

**Affiliations:** 1 Eye Institute of Shandong First Medical University, State Key Laboratory Cultivation Base, Shandong Provincial Key Laboratory of Ophthalmology, Qingdao, Shandong China; 2 Qingdao Eye Hospital of Shandong First Medical University, Qingdao, Shandong China; 3 Eye Hospital of Shandong First Medical University, Jinan, Shandong China; 4 School of Ophthalmology, Shandong First Medical University, Jinan, Shandong China

**Keywords:** Mechanisms of disease, Transcriptomics

## Abstract

Keratoconus is a common ectatic corneal disorder in adolescents and young adults that can lead to progressive visual impairment or even legal blindness. Despite the high prevalence, its etiology is not fully understood. In this study, we performed single-cell RNA sequencing (scRNA-Seq) analysis on 39,214 cells from central corneas of patients with keratoconus and healthy individuals, to define the involvement of each cell type during disease progression. We confirmed the central role of corneal stromal cells in this disease, where dysregulation of collagen and extracellular matrix (ECM) occurred. Differential gene expression and histological analyses revealed two potential novel markers for keratoconus stromal cells, namely *CTSD* and *CTSK*. Intriguingly, we detected elevated levels of *YAP1* and *TEAD1*, the master regulators of biomechanical homeostasis, in keratoconus stromal cells. Cyclical mechanical experiments implicated the mechanical stretch in prompting protease production in corneal stromal cells during keratoconus progression. In the epithelial cells of keratoconus corneas, we observed reduced basal cells and abnormally differentiated superficial cells, unraveling the corneal epithelial lesions that were usually neglected in clinical diagnosis. In addition, several elevated cytokines in immune cells of keratoconus samples supported the involvement of inflammatory response in the progression of keratoconus. Finally, we revealed the dysregulated cell-cell communications in keratoconus, and found that only few ligand-receptor interactions were gained but a large fraction of interactional pairs was erased in keratoconus, especially for those related to protease inhibition and anti-inflammatory process. Taken together, this study facilitates the understanding of molecular mechanisms underlying keratoconus pathogenesis, providing insights into keratoconus diagnosis and potential interventions.

## Introduction

Keratoconus, which can lead to visual impairment or even legal blindness^1,2^, is a common progressive corneal disorder characterized by thinning and asymmetrical conical protrusion of the cornea in young people^3,4^. Keratoconus is one of the leading indications for corneal transplantation surgery worldwide^5–7^, with a prevalence of 1/2000 in the general population and even higher in young adults^3,4,8^. Keratoconus was classically defined as a progressive, non-inflammatory disease^9^. Clinically, the central corneal stroma undergoes gradually thinning and loss of structural integrity that leads to corneal bulging, which gives the cornea a typical cone shape appearance in patients with keratoconus^10^. Keratoconus that progresses into the most severe stage manifests with excessive ectasia, thinning, scarring and thereby significantly impairs the vision, and corneal transplantation is considered as the last resort^1^. The development of keratoconus involves complex interactions between genetic and environmental factors^11,12^, while their specific contributions to this disease are mostly unknown and likely to be variable. Currently, no ideal animal model for keratoconus is available. Despite a great number of studies, the etiology of keratoconus is still poorly understood. Thus, a detailed exploration of its pathophysiological changes and molecular mechanisms is urgently needed.

The corneal stroma, which comprises the bulk of the corneal thickness, is a highly ordered network of collagen fibrils and extracellular matrix (ECM)^13,14^. Early clues suggested that the culprit in keratoconus is perhaps collagen degradation due to elevated gelatinases, metalloproteinases (MMPs) and catalases in keratoconus stromal cells^15,16^, which was traditionally considered to be the main explanation for keratoconus pathogenesis. However, these findings were mainly determined by histological or biochemical examinations, and merely represented partially pathological changes. Several contemporary studies have also investigated the genomic, transcriptomic or proteomic alterations in keratoconus samples^6,17,18^, though stroma degradation were spotted, more multifaceted and precise signals in cell type-specific manners were diluted because of the limitation of bulk-input methods. In addition, previous studies demonstrated that keratoconus is often accompanied by altered biomechanical properties of the cornea, which can be an indicator of healthy and keratoconus eyes^19–23^. Multivariate analysis of risk factors for keratoconus determined that eye rubbing was the most significant predictor for the progression of keratoconus^24,25^. Vigorous eye rubbing was shown sharply increased force to corneas and a consistent association with keratoconus was observed^24,26^. However, the role of mechanical stretch in keratoconus progression and the pathological relevance between mechanical stretch and corneal stroma degradation remain elusive, and thus in-depth investigations of specific and explicit molecular mechanisms need to be conducted.

The cornea is one of the only few transparent tissues of the body^27^. Though its cellular composition is relatively uncomplicated, problems with either cell type might cause serious consequences. And the heterogeneity for each cell type remains largely unknown. Single-cell RNA sequencing (scRNA-Seq) can help us access molecular profiles into disease at unprecedented resolution. scRNA-Seq has been demonstrated in multiple ocular tissues including retina^28–30^, sclera^31^ and limbal/corneal epithelium^32,33^. However, the single-cell landscape of corneas with keratoconus remains to be systematically depicted. In this study, we obtained central cornea tissues from patients with keratoconus and healthy individuals, and elucidated the cell type-specific transcriptional alterations in keratoconus. In corneal stromal cells, we identified two novel keratoconus-related markers, namely *cathepsin D* (*CTSD*) and *cathepsin K* (*CTSK*). We also detected the up-regulation of the mechano-transducer *YES-associated protein 1* (*YAP1*) as well as its cooperator *TEA domain transcription factor* (*TEAD1*) in keratoconus stromal cells. Further cyclical mechanical experiments revealed that several protease genes (including *MMP1*, *MMP3*, *CTSD* and *CTSK*) can be induced by stretch, indicating the inductive role of mechanical stretch in keratoconus pathogenesis. These results orchestrated the pathogenic role of biomechanics-enzymes axis in keratoconus progress, providing explanations for the clinical phenomenon that mechanical stimuli can lead to the onset and exacerbation of keratoconus symptom. In the corneal epithelium, the reduced basal cells and abnormally differentiated superficial cells implied the corneal epithelial lesions that were easily missed during clinical diagnosis. Besides, a range of elevated cytokines in immune cells of keratoconus samples supported the participation of inflammation in keratoconus progress. Furthermore, the dysregulated cell-cell communications in keratoconus were comprehensively compiled, revealing the erased ligand-receptor interactions in keratoconus, especially for those related to protease inhibiting and anti-inflammatory processes. Overall, this study provided valuable resources for understanding keratoconus pathogenesis, offering further insights to improve preventative and therapeutic strategies for this disease.

## Results

### Single-cell transcriptomic profiling of corneas from keratoconus patients and healthy individuals

**Fig. 1:**
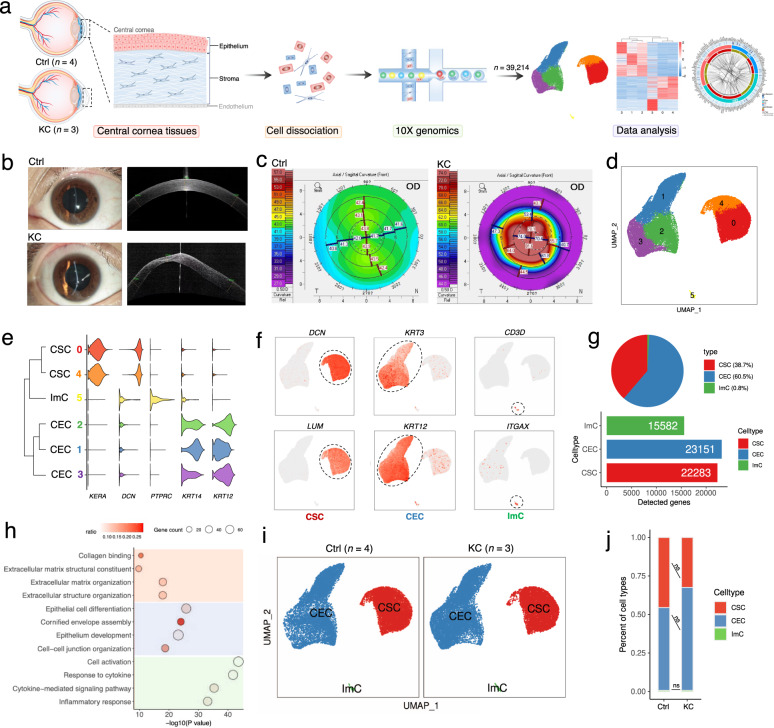
Overview of cellular compositions of KC and Ctrl human corneas delineated by scRNA-Seq analysis. **a** Overview of the experimental workflow in this study. In the schematic of diagram of the human cornea, the corneal epithelium and stroma (labeled in black) from the central cornea were subjected to downstream experiments, and the corneal endothelium (labeled in gray) was excluded (see Materials and Methods). **b**, **c** Anterior segment OCT (**b**) and Scheimpflug optical cross-sectional analysis (**c**) showed typical symptoms of keratoconus cornea. **d** UMAP representation of human corneal cells colored into 6 distinct clusters. **e**, **f** Expression levels (**e**) and distribution (**f**) of well-known representative cell markers across clusters. **g** The cell type proportions (top panel) and the number of detected genes per cell type (bottom panel). **h** Representative GO terms of specifically expressed genes in each cell type. **i** UMAP plot of human corneal cells colored by three major cell types in KC and Ctrl groups. **j** Bar plot representing the differences in relative proportion of major cell types between Ctrl and KC samples. ns, no significance (Student’s *t*-test). KC keratoconus; Ctrl control; CSC corneal stromal cell; CEC corneal epithelial cell; ImC immune cell.

To comprehensively understand the cellular diversity and molecular signatures of the human cornea from keratoconus and healthy control individuals, we isolated corneal cells from three central cornea tissues (without endothelium) of keratoconus patients after deep anterior lamellar keratoplasty (DALK) surgeries^34,35^ and four control samples from healthy donors (Supplementary Table S1, see Materials and Methods for details). Tissues from each sample were dissociated and subjected to 10x Genomics platforms for scRNA-Seq, separately (Fig. 1a). The severity score of all of the keratoconus samples was 5 according to the Keratoconus Severity Score (KSS) ranking scheme^36^. As expected, anterior segment optical coherence tomography (OCT) and Scheimpflug optical cross-sectional analysis showed the pathobiology of keratoconus including a protrusion and thinning at the top of the cone, sagittal curvature and posterior elevation subtraction (Fig. 1b, c). These evident changes in the central cornea prompted us to investigate underlying mechanisms of keratoconus pathogenesis.

Following strict quality control steps and doublet removal, a total of 39,214 cells were obtained from all samples, with an average of 3339 genes and 18,247 transcripts per cell (Supplementary Fig. S1a, b). Of these, 20,312 cells originated from keratoconus corneas (abbreviated as “KC” in the following), and 18,902 cells were from healthy corneas (abbreviated as “Ctrl”). Upon unsupervised clustering, we divided all cells into 6 clusters (Fig. 1d) with distinct transcriptional signals (Supplementary Fig. S1c and Table S2) by Uniform Manifold Approximation and Projection (UMAP) embedding, and three primary cell types were annotated according to classical specific markers (Fig. 1e, f), namely corneal epithelial cells (CECs), corneal stromal cells (CSCs), and a few immune cells (ImCs). The cell type composition, as well as the number of detected genes in each cell type were represented in Fig. 1g. For the distribution of cell types in KC and Ctrl samples, separately, no significant differences or extra clusters were observed, indicating a physiological difference-based cell clustering rather than variations caused by technical batch or individual differences (Supplementary Fig. S1d). Specifically, the most abundant of all cells (60.5%) were CECs, which were identified based on classical epithelium-specific markers, such as *KRT3*, *KRT12* and *KRT14*^32,33^ (Fig. 1e, f). Clusters corresponding to corneal CSCs (38.7%) were annotated according to *DCN*, *KERA* and *LUM*^32,33^. ImCs (0.8%), that were identified by *PTPRC* (also known as CD45), contained T cells (*CD3D*^*+*^) and dendritic cells (*ITGAX*^*+*^) (Fig. 1e, f). Taken together, we revealed the cellular composition of the central corneas from keratoconus patients and healthy individuals, and provided a comprehensive representation of corneal cells for further studies of keratoconus pathogenesis. Besides the prominent cell populations, several rare populations with a small percentage, such as ImCs, that were difficult to be captured by traditional strategies, were effectively arrested and annotated in our data. Furthermore, biological functions of differentially expressed genes (DEGs) for each cell type were analyzed to decipher their unique gene expression signatures (Fig. 1h; Supplementary Table S1). To be specific, gene ontology (GO) terms including “collagen binding”, “extracellular matrix structural constitute” and “extracellular matrix organization” were enriched for CSCs. Terms corresponding to CECs included “epithelial cell differentiation”, “cornified envelope assembly” and “epidermis development”. Terms involving “response to cytokine”, “cytokine-mediate signaling pathway” and “inflammatory response” were enriched for ImCs. We also surveyed the proportions of these three cell types across Ctrl and KC samples, and no significant disparity was observed (Fig. 1i, j; and Supplementary Fig. S1e), suggesting that abnormal gene expression in these cells may be more important than changes in their proportions in the pathological mechanism of keratoconus. Thus, the cell type-specific alterations in keratoconus need to be explored.

### Cell type-specific transcriptional alterations in keratoconus corneas

**Fig. 2:**
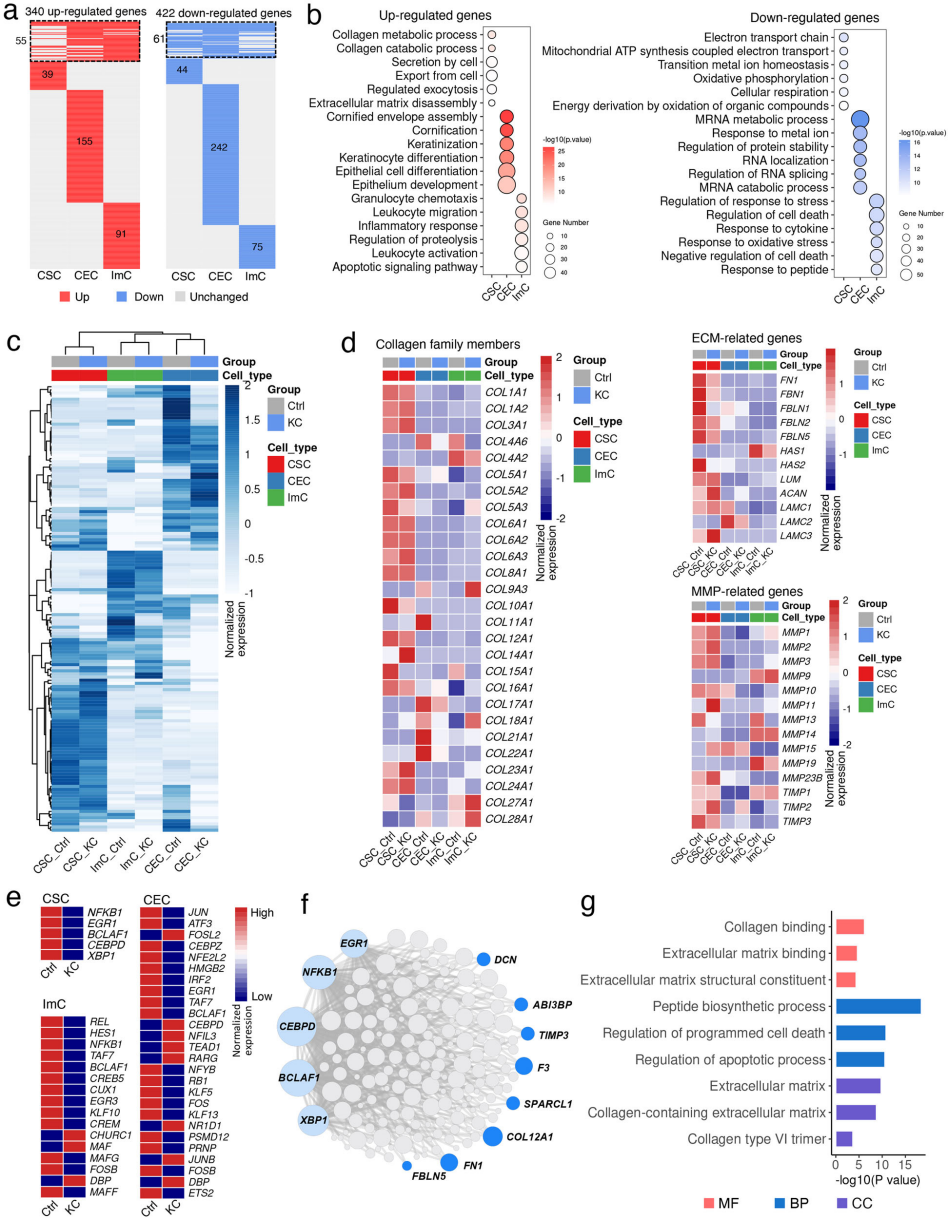
Changes in transcriptional profiles and regulatory networks of each cell type in keratoconus corneas. **a** Heatmaps showing the distribution of up-regulated (red) and down-regulated (blue) DEGs in each cell type in KC samples compared with those in Ctrl samples, and genes not differentially expressed are in gray. Rows represent genes and columns denote cell types. The upper part within dashed boxes indicates the DEGs shared by at least two cell types, while the lower panel indicated the unique DEGs for each cell type. The numbers of DEGs are annotated on the plots. **b** Representative GO terms of up-regulated (red) and down-regulated (blue) DEGs for each cell type. **c** Cell type-specific gene expression profile of keratoconus-associated genes. The value for each gene is the row-scaled Z score. **d** The expression changes of representative collagen family members, ECM and matrix metalloproteinase-related genes between KC and Ctrl samples in each cell type. **e** Heatmap showing TF expression discrepancy between Ctrl and KC in each cell type. **f** Regulatory network visualizing TFs identified in CSCs and their target genes predicted by SCENIC. TFs are colored in light blue and representative target genes down-regulated in keratoconus are in dark blue. The node sizes are positively correlated to the edge counts for each node. **g** Representative GO enrichment terms for genes targeted by TFs identified in CSCs.

We compared the transcriptional signatures of each cell type in KC and Ctrl samples. DEGs between the two groups were identified in CSCs, CECs and ImCs, separately. A total of 340 up-regulated and 422 down-regulated genes were differentially expressed in at least one cell type (Fig. 2a). Only ~15% of DEGs were shared by at least two cell types, and the majority of DEGs exhibited in a cell type-specific manner (Fig. 2a). For up-regulated genes, DEGs in CSCs were involved in collagen metabolic process and ECM disassembly, providing the theoretical explanation for the degradation and stromal thinning in keratoconus^13^. While in CECs, terms including “cornification” and “epithelial cell differentiation” were enriched. DEGs in ImCs were enriched for immune-related processes such as leukocytes migration and inflammatory responses (Fig. 2b). By comparison, the biological functions annotated for down-regulated DEGs in three cell types were associated with electron transport chain, mRNA metabolic progress and response to stress, separately (Fig. 2b). Then we used our scRNA-Seq data to map the cell type-specific expression patterns of 136 keratoconus-associated genes downloaded from OMIM Clinical Synopsis (https://www.omim.org) (Fig. 2c). We found that the highest percentage of genes were enriched in CSCs, followed by CECs with an even more divergent expression pattern between groups. Although the proportion of ImCs was much lower in the entire tissue, quite a few DEGs (~20%) were derived from these cells, implying the role of immunocytes in keratoconus development that can not be ignored^37^. Here our data implied that keratoconus is a complex disease that involves multiple cell types, and cell type-specific changes and mechanisms are needed to be further investigated.

We then accessed the cell type-specific expression patterns of known genes related to keratoconus, such as *COL5A1*, encoding the α1 chain of type V collagen, a structural protein of the ECM^38^; and *MMP1/3*, members of matrix metalloproteinases^15,16,18^. We profiled relevant gene family members across cell types in both Ctrl and KC samples, and found that almost all collagen genes were enriched in stromal cells and several genes were down-regulated in KC samples, such as *COL5A1*, *COL5A3*, *COL10A1* and *COL15A1* (Fig. 2d), which explained the decreased collagen lamellae and fibers in keratoconus^39^. Notably, beyond collagens, the expression of ECM molecules such as glycoproteins and proteoglycans had extensive changes. For example, genes encoding ECM molecules (such as *FN1*, *FBN1* and *FBLN1*) were extensively down-regulated in KC stromal cells (Fig. 2d). For MMP-related genes, several up-regulated members were detected in KC stromal cells, in addition to previously reported *MMP1* and *MMP3*, other members such as *MMP11*, *MMP23B* and *MMP15* were also detected; and the metallopeptidase inhibitor *TIMP3* decreased in KC stromal cells. Interestingly, we noted that the transcriptional changes of collagen, ECM- and MMP-related genes were not restricted to stromal cells, for instance, *COL4A6*, *COL17A1*, *COL21A1*, *COL22A1* and *LAMC2* were down-regulated in CECs; down-regulated *HAS1* and up-regulated *MMP9* were detected in ImCs (Fig. 2d). Therefore, all cell types may play vital roles in ECM dysregulation during keratoconus progression.

To assess the expression status of transcription factors (TFs) in the healthy cornea and identify potential TFs modulating the differential transcriptional signatures in keratoconus, we performed the single-cell regulatory network inference and clustering (SCENIC) analysis in each cell population^40^, and predicted the cell type-specific TFs in KC and Ctrl samples (Fig. 2e). We noted that five TFs including *NFKB1*, *EGR1*, *BCLAF1*, *CEBPD* and *XBP1* were extensively decreased in KC stromal cells (Fig. 2e, Supplementary Fig. S2). And some of their target genes, such as *FN1*, *COL12A1*, *TIMP3* and *FBLN5*, were associated with ECM and also down-regulated in keratoconus (Fig. 2f). GO analysis showed that predicted target genes by these TFs were enriched for extracellular matrix, collagen, peptidase synthesis and cell apoptosis (Fig. 2g). For instance, *EGR1* was reported to be recruited to the promoters of *Col1a1* and *Col2a1* in postnatal mouse tendons^41^, and silencing *EGR1* could significantly suppress *Col1a1* and *Col1a2*^42^. For TFs identified in CECs, we detected an up-regulated TF *TEAD1*, as a mechano-responsive gene, plays an important role in force-induced transcriptional regulation^43^. Several TFs were down-regulated in ImCs, such as *REL*, its pro-survival, anti-oxidative stress and anti-inflammation roles were reported in Parkinson’s progression^44^; while for *DBP*, which was reported to enhanced a pro-inflammatory state through increasing the release of TNF-α and IL-1β^45^, were up-regulated in CECs and ImCs of keratoconus. In summary, we identified the dysregulation of candidate TFs in each cell type, suggesting their potential regulatory roles in keratoconus progression.

### Transcriptional changes in keratoconus stromal cells and the involvement of mechanical stretch

**Fig. 3:**
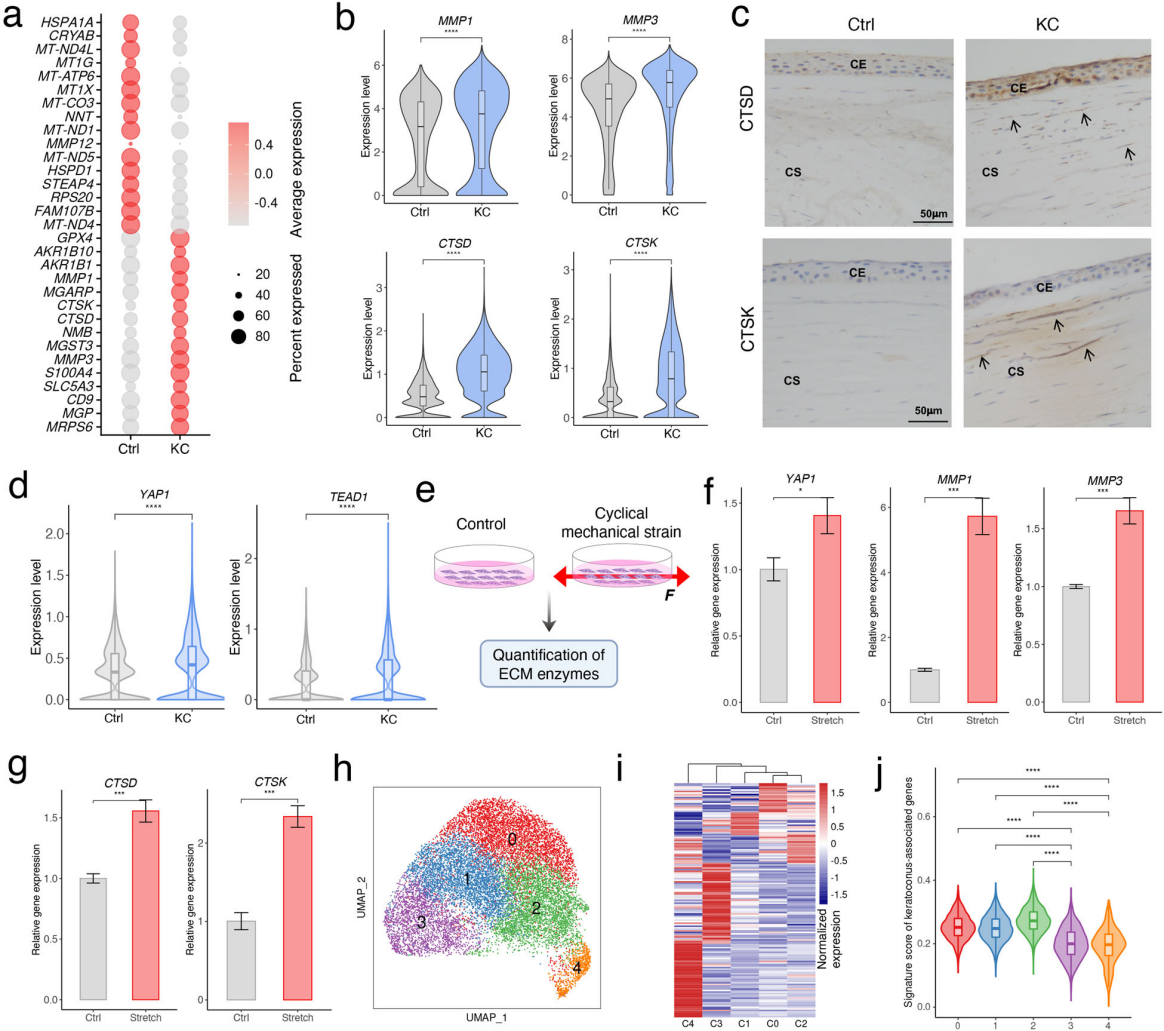
Transcriptional alterations and heterogeneous analysis of CSCs in KC and Ctrl samples. **a** Dot plot showing top 15 up- and down-regulated genes in keratoconus CSCs than that in Ctrl samples. **b** Expression changes of *MMP1*, *MMP3*, *CTSD* and *CTSK* in keratoconus CSCs. **c** Immunohistochemical analysis showing the elevated level of CTSD and CTSK in keratoconus stroma. **d** Expression changes of *YAP1* and *TEAD1* in keratoconus CSCs. ****P < 0.0001 (two-sided Wilcoxon rank-sum test). **e** Schematic diagram of cyclical mechanical experiments on cell lines. The mechanical stretch exerted on cells was indicated by red arrow. **f** Expression of *YAP1*, *MMP1* and *MMP3* in HTK cells quantified by RT-qPCR. *P < 0.05, ***P < 0.001 (Student’s *t*-test). **g** Quantification of *CTSD* and *CTSK* in HTK cells by RT-qPCR. ***P < 0.001 (Student’s *t*-test). **h** UMAP clustering for corneal stromal cells colored by different subtypes. **i** Heatmap of expression signals of top specifically expressed gene in each subtype of CSCs. The value for each gene is the row-scaled Z score. **j** Gene scoring analysis across CSC subtypes using keratoconus-associated genes. ****P < 0.0001 (two-sided Wilcoxon rank-sum test).

Based on DEGs of CSCs between KC and Ctrl samples (Fig. 2a), we selected several top genes for further studies (Fig. 3a). Genes up-regulated in KC stromal cells included *MMP1* and *MMP3* (Fig. 3a, b), which were reported to be able to degrade fibrillar corneal collagens and play a role in stromal thinning in keratoconus^46^. Here we identified two novel potential KC-related enzymes (Fig. 3a, b), including *CTSD*, which encodes an aspartic protease involved in the regulation of proteolytic activity of lysosomes^47^; and *CTSK*, which encodes a lysosomal cysteine protease that is essential for the degradation of proteins in bone matrix including collagen type I^48^. Compared to *MMP1* and *MMP3*, *CTSD* and *CTSK* differentially expressed between KC and Ctrl samples with a higher specificity (indicated as higher pct.1/pct.2 ratio) (Supplementary Fig. S3a). Consistent with the detected changes in mRNA levels (Fig. 3b), immunohistochemical analyses confirmed increased CTSD and CTSK protein levels in keratoconus stromal cells (Fig. 3c). As mentioned, up-regulated genes in CSCs were enriched in collagen metabolic process and ECM disassembly, the contributions of these dysregulated proteases should not be ignored in the stromal degradation of keratoconus cornea. Herein, we provided new biomarkers and potential targets for the diagnosis and treatment for keratoconus.

Several studies have suggested that many diseases were associated with an imbalance of ECM synthesis and degradation with the involvement of mechanical factors. Previous studies have shown that YAP is induced in response to mechanical stimuli^49,50^, and TEAD can mediate YAP-dependent gene induction and growth control^51^. Keratoconus is usually accompanied by altered biomechanical properties of the cornea^23,52,53^. To test whether the expression of YAP and TEAD changed in keratoconus, we surveyed their expression level in our data, and found that both *YAP1* and *TEAD1* elevated in keratoconus stromal cells, implying the response of keratoconus stromal cells to mechanical stretch (Fig. 3d). Several studies suggested that mechanical stimulation is involved in the regulation of MMPs in some organs, such as human bladder smooth muscle cells^54^, musculoskeletal progenitors^55^, or even some ocular tissues, including sclera fibroblasts^56^, trabecular meshwork^57^ and lamina cribrosa cells^58^. To further investigate the role of mechanical stretch in altered transcriptional signatures of keratoconus stromal cells, we performed cyclical mechanical stimulation on a human stromal keratocyte cell line, HTK cells (Fig. 3e). Strikingly, we detected a significant increase of *YAP1* (Fig. 3f), suggesting the activated cellular response to mechanical stretch; and elevated *MMP1* and *MMP3* after mechanical stimulation were observed (Fig. 3f). Besides, we also found that cyclical mechanical stretch increased the expression of *CTSD* and *CTSK* in HTK cells (Fig. 3g), suggesting the perturbation in cellular homeostasis of ECM turnover enzymes caused by mechanical stimulation. Overall, these observations provided a plausible self-contained homeostatic mechanism that explained the aggravation of mechanical stimulation on keratoconus progression by triggering a variety of ECM enzyme expressions, though further studies were needed to fully elucidate its step-by-step mechanisms.

As reported, the pathogenesis of keratoconus was along with metabolic changes, including pentose phosphate pathway, oxidative stress, and glycolysis^59,60^. We next wondered whether and which metabolic process altered in keratoconus stromal cells. Here we scored KC and Ctrl CSCs for their expression of oxidative phosphorylation and glycolysis. Notably, we observed significantly increased oxidative phosphorylation and glycolysis score in KC samples (Supplementary Fig. S3b). Another recent study has reported that metabolic responses such as glycolysis were coupled to cell mechanics^61^, which enlightened that the relevance among metabolic process, cell mechanics and keratoconus pathogenesis might be a much more interesting issue that deserved in-depth exploration. Besides, the score of DNA damage displayed a striking upregulation in KC samples (Supplementary Fig. S3b), which coincided with the previous report that keratoconus corneas exhibit more mitochondrial DNA damage than healthy corneas^62,63^, involving an imbalance of redox homeostasis in this disease.

Currently, the understanding of cellular heterogeneity on corneal stroma was limited. To explore the heterogeneous changes in keratoconus corneas, we divided CSCs into five subpopulations (C0 to C4, Fig. 3h). All these subtypes are uniformly distributed in Ctrl and KC samples with similar proportions (Supplementary Fig. S4a, b). When we identified highly expressed genes for each subtype (Fig. 3i; Supplementary Fig. S4c, Table S3), GO analysis revealed that biological process related to ECM was enriched in C0 and C2, term of response to hypoxia was enriched in C1, and terms of response to unfolded protein was enriched in C3 and C4 (Supplementary Fig. S4d), suggesting a heterogeneity in function or state of CSCs. We then evaluated the subtype-specific expression of the keratoconus-related enzymes identified in this study, and found that *CTSD* was specifically and highly expressed in keratoconus across all subtypes, while *CTSK* was mainly differentially enriched in C0-C2 cells (Supplementary Fig. S5a). Furthermore, based on keratoconus-associated genes, we used our single-cell transcriptomic data to relate the keratoconus association with patterns of cell-type specific expression. And we found that higher percentage of associated genes were preferentially enriched in C0-C2 (Supplementary Fig. S5b, c), and C0-C2 cells displayed higher signature scores of keratoconus-associated genes compared to C3-C4 cells (Fig. 3j), suggesting that C0-C2 subtypes are likely to be more associated with the susceptibility of keratoconus.

### Abnormally differentiated corneal superficial cells in keratoconus samples

**Fig. 4:**
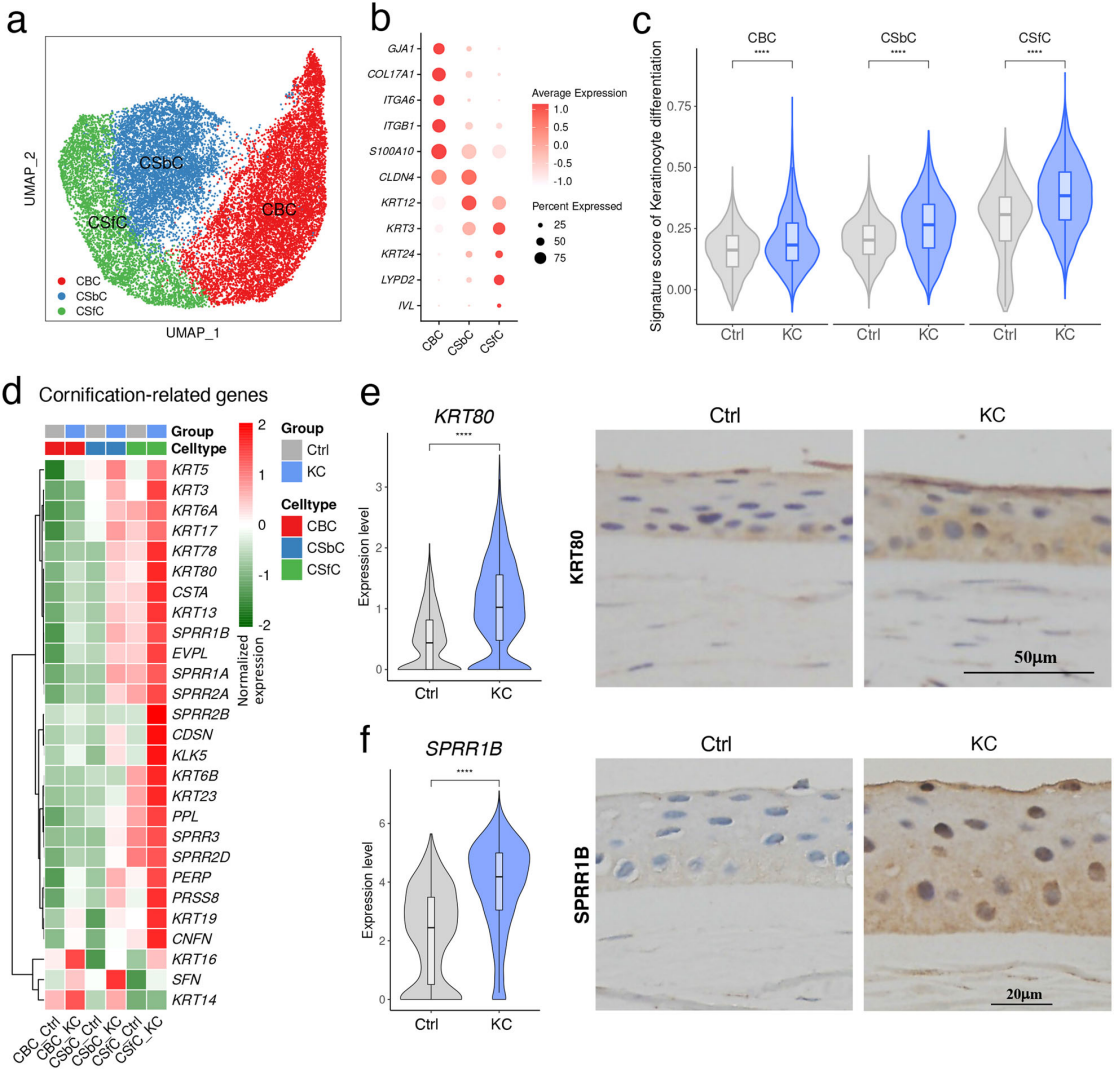
Heterogeneous transcriptional changes in keratoconus corneal epithelium. **a** UMAP plot of corneal epithelial cells colored by annotated cell types. **b** Dot plot showing expression patterns of representative markers for each cell type. **c** Gene scoring analysis using keratinocyte differentiation-related genes. ****P < 0.0001 (two-sided Wilcoxon rank-sum test). **d** Expression profiling for cornification-related genes across cell subtypes and groups. **e**, **f** Expression level of *KRT80* (**e**) and *SPRR1B* (**f**) in Ctrl and KC superficial cells (left panel), and immunohistochemical analysis confirmed their elevated expression in KC samples (right panel). ****P < 0.0001 (two-sided Wilcoxon rank-sum test).

To deeply dissect the cellular and transcriptional changes in keratoconus CECs, we then performed unsupervised sub-clustering on these cells. Accordingly, three subclusters with distinct transcriptional signatures were observed in both Ctrl and KC samples (Supplementary Fig. S6a, b). Well-known specific markers, such as *GJA1* for corneal basal cells (CBCs)^64^, *KRT3* for corneal suprabasal cells (CSbCs, also known as wing cells)^65^, *IVL* and *LYPD2* for corneal superficial cells (CSfCs, also known as squamous cells)^32^, were used to determine the cellular identity of these clusters (Fig. 4a, b, Supplementary Table S4). We then utilized the pseudotime algorithm SCORPIUS^66,67^ to reconstruct the differentiation trajectory of these cell populations, and observed a predicted trajectory from CBCs to CSfCs, and CSbCs fell in between (Supplementary Fig. S6c, d), in line with the anatomical and developmental characteristics of corneal epithelium^68,69^. To understand the changes of corneal epithelium in keratoconus, we first surveyed the proportional variance of each cell type, and decreased basal cells were observed (Supplementary Fig. S6e). Intriguingly, when we profiled the cell cycle state of each cell, cells in S phase were commonly decreased, and cells in G2/M phase were increased by approximately 1.5-fold in keratoconus, indicating diminishing quiescence and activated cell division in keratoconus (Supplementary Fig. S6f). According to the intensive signals of keratinocyte differentiation and cornification in keratoconus (Fig. 2b), we scored three epithelial cell types in KC and Ctrl samples with keratinocyte differentiation-related genes, and all cell types demonstrated significant higher differentiation score in keratoconus, especially for CSfCs that with the greatest change (Fig. 4c). Then we profiled the cell type-specific expression of cornification- and differentiation-related genes, we found that stronger signature of these two gene sets were enriched in KC samples, especially in CSfCs (Fig. 4d; Supplementary Fig. S6g). Among these genes, a highly and specifically expressed gene in keratoconus CSfCs, *KRT80* (Fig. 4d, e; Supplementary Fig. S6g), which was related to advanced tissue or cell differentiation^70^. Immunohistochemical staining of KRT80 confirmed the hyper-differentiation signal of corneal superficial layer in keratoconus (Fig. 4e). In addition, we noted another significantly increased gene in KC samples, *SPRR1B* (Fig. 4f), a valid biomarker for the study of superficial cell differentiation and squamous metaplasia in the cornea^71^. Squamous metaplasia is an abnormal epithelial differentiation represented on the ocular surface with the loss of cornea-specific keratin K12 and the emergence of epidermis-specific keratin K10^72,73^. Interestingly, previous studies revealed the squamous metaplasia in the conjunctival epithelium of keratoconus^73^, but the metaplastic squamous epithelia in central cornea were not reported. Besides the elevated SPRR1B level, K10^+^ cells as well as the reduced expression of K12 and PAX6 were observed in keratoconus epithelium (Supplementary Fig. S7), implying the lesion of corneal epithelial squamous metaplasia in keratoconus^74^. Taken together, our data demonstrated that in keratoconus patients, corneal basal cells reduced and superficial cells showed active differentiation, increased cornification and squamous metaplasia signal, indicating cell type-specific state shifting in keratoconus corneal epithelium that cannot be ignored in clinical diagnosis and treatment.

### Increased cytokines unveiled the contribution of immunocytes in keratoconus pathophysiology

**Fig. 5:**
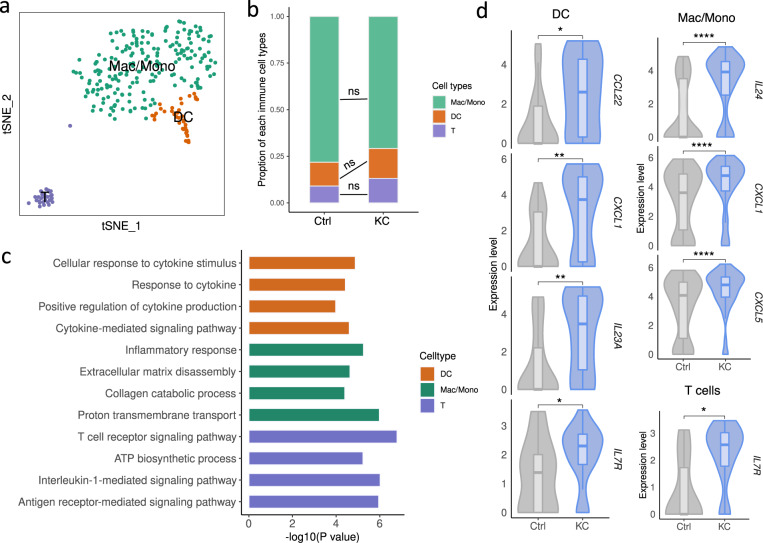
Inflammatory signals in keratoconus corneas contributed by immune cells. **a** t-SNE plot showing three immune cell types identified in KC and Ctrl samples. **b** Proportion of each immune cell type in KC and Ctrl samples. ns, no significance (Student’s *t*-test). **c** Representative GO terms of keratoconus-upregulated genes in each immune cell type. **d** Violin plots showing representative differentially expressed cytokines in keratoconus immune cells. *P < 0.05, **P < 0.001, ****P < 0.0001 (two-sided Wilcoxon rank-sum test).

Keratoconus was traditionally classified as a non-inflammatory disease^9^. Nonetheless, emerging evidence has supported certain inflammatory properties involved in keratoconus^37,75^. For instance, the majority of studies in the tears of patients with keratoconus have shown increased levels of IL-6^76^. However, a comprehensive detection of a variety of cytokines and the source of cytokine secretion remain unclear. To address this controversial issue at the single-cell level, we first surveyed the predominant source of several reported cytokines increased in keratoconus samples, including IL1, IL6, IL10, TNF-α and TGF-β^37,75,76^. In our study, we observed that except for TGF-β, almost all of these cytokines were specifically enriched in ImCs (Supplementary Fig. S8). Then we focused on the cellular alterations in immune cell types as a starting point. After unsupervised sub-clustering by the t-distributed stochastic neighbor embedding (t-SNE)^77^, we identified macrophages/monocytes (Mac/Mono), dendritic cells (DC), and T cells (T) in both KC and Ctrl samples (Fig. 5a; Supplementary Fig. S9a–c) based on specific markers. No obvious proportional variation of each immune cell type was detected between KC and Ctrl samples (Fig. 5b). Therefore, we performed the differential expression analysis in a cell type-specific manner. Strikingly, GO analysis of upregulated genes in KC showed increased inflammatory responses and extracellular matrix disassembly in Mac/Mono; terms of response to cytokine were enriched in DCs; while T cells were enriched for T cell receptor signaling pathway and ATP biosynthetic process (Fig. 5c). We then examined a series of inflammatory factors in each immune cell. Notably, multiple interleukins (such as *IL23A*) and chemokines (such as *CXCL1*) significantly elevated in keratoconus DCs, indicating activated immune response in DCs during keratoconus pathogenesis (Fig. 5d). Besides, macrophages/monocytes and T cells of KC showed several elevated cytokines as well. Collectively, these findings were in accord with previous observations that increased cytokines could be detected in keratoconus specimen^37^. This study provided supporting evidence for the inflammatory properties of keratoconus with precise cellular identity at unprecedented resolution.

### Aberrant cell–cell communications in keratoconus corneas

**Fig. 6:**
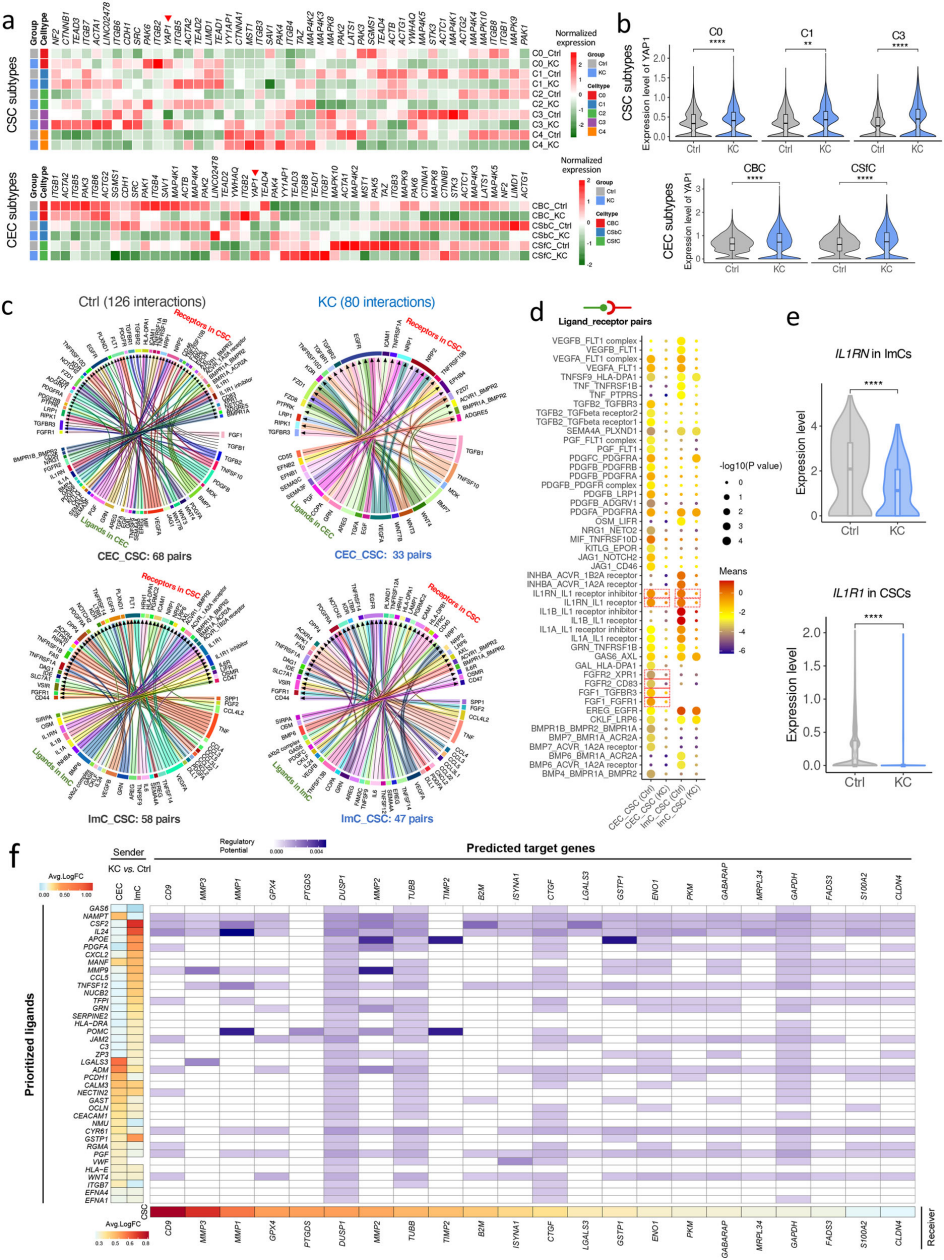
Keratoconus-related changes in cell–cell communications. **a** Expression profiles for YAP signaling pathway components in CSC and CEC subclusters. **b** Expression changes of YAP1 in CSC and CEC subclusters of keratoconus samples. **P < 0.01, ****P < 0.0001 (two-sided Wilcoxon rank-sum test). **c** Chord diagram of cellular interactions between ImCs/CECs and CSCs from Ctrl and KC samples, separately. The number of interaction pairs and cell types are annotated. **d** Dot plot of erased ligand-receptor interactions associated with CSCs in KC samples compared to that in Ctrl samples. CSCs express receptors and receive ligand signals from CECs/ImCs. The row represents a ligand-receptor pair and the column defines a cell-cell interaction. The samples of Ctrl or KC are annotated in the brackets. *P* values and means was calculated by CellphoneDB pipeline. The dot size reflects the *P* values for cell type-specificity, and the dot color denotes the mean of the average ligand-receptor expression in the corresponding interacting cell types. The pairs mentioned in the text are in dashed boxes. **e** Bar graph of the expression of *IL1RN* in ImCs and *IL1R1* in CSCs. **f** NicheNet interaction heatmap between ImCs/CECs and CSCs in KC samples. Left, heatmap of average log_2_FC of the top predicted ligands expression between KC and Ctrl samples for CECs/ImCs. Bottom, heatmap of average log_2_FC of ligand-matched targets expression between KC and Ctrl samples for CSCs. Middle, heatmap of predicted ligand-target regulatory potential.

As reported, YAP signaling pathway was closely involved in mechanotransduction signaling sensing when the extracellular mechanical microenvironment changes^78^. Keratoconus has abnormal biomechanical properties and the values of corneal biomechanical measurements are significantly lower in keratoconus eyes than in healthy eyes^79^. Considering the mechanical properties of keratoconus, we globally profiled the expression of genes harbored in YAP signaling pathway. On the whole, compared to control samples, we observed that a substantial portion of components were down-regulated in keratoconus, in both CSCs and CEC subtypes (Fig. 6a). And we noted that, *YAP1*, the key activator and central component of this pathway, was significantly increased in C0, C1 and C3 subclusters of CSCs, as well as CBCs and CSfCs (Fig. 6b). Immunohistochemical staining confirmed the elevated expression of YAP1 in both corneal stroma and epithelium (Supplementary Fig. S10a).

Earlier studies indicated that stromal-epithelial and stromal-epithelial-immune interactions were key determinants of corneal function and homeostasis^80,81^. Bi-directional communications occur in a highly coordinated manner between corneal cells during normal development, wound healing and disease^80^. However, the systematic studying of altered cell–cell interactions in keratoconus is still lacking. In order to decipher the ligand-receptor interactions among corneal cells, we performed cell–cell interaction analysis (CellPhoneDB)^82^. A large number of ligand-receptor pairs among all cell types were identified in Ctrl and KC samples, separately (Supplementary Fig. S10b; Tables S5, S6). We then attempted to detect altered cell–cell communications in KC compared to Ctrl samples, found that only few interactions were gained in KC, but a large fraction of pairs was erased, especially between CECs and CSCs/ImCs, and the auto-regulation in CECs (Supplementary Fig. S10c). All erased interaction pairs among CECs, CSCs and ImCs in KC samples were plotted in Supplementary Fig. S10d. For example, ANXA1-FPR1 system is potent effective mediators in anti-inflammatory processes^83^, and their interaction was lost between CECs and ImCs in KC samples; IL1RN-IL1R1 are natural anti-inflammatory cytokines^84^, and their interaction were eliminated between CECs and CSCs in KC samples. All these findings were consistent with the increased inflammatory signals in keratoconus ImCs as aforementioned. Besides, TIMP1 (with its ligand EGFR) is secreted glycoprotein that blocks MMP activity to maintain them in an inactive state^85^, which was erased between CSCs and CECs in KC samples, in line with the increased MMP1/3 level in keratoconus stromal cells. Herein, each cell type in cornea participated in cell–cell communications with high levels, and GO analysis of erased interactions among keratoconus cells revealed aberrant cell–cell interactional links involved in multiple pivotal biological processes, such as tissue morphogenesis, regulation of cell–cell adhesion, protein metabolic process, and quite a few important signaling pathways were also included (Supplementary Fig. S10e). Considering that the stroma is the major site where thinning and collagen degradation occurs during keratoconus progression^86^, we then focus on the exploration of the changed interaction links associated with CSCs. 126 interactions in Ctrl and 80 in KC samples were observed (Fig. 6c), consistent with the overall reduction of interactional links in KC samples. Notably, several erased interactions between CECs and CSCs, such as FGFR2–XPR1, FGFR2–CD83, FGF1–TGFBR3, FGF1–TGFBR3, were associated with the fibroblast growth factor signaling pathway (Fig. 6d). The erased interactions between ImCs and CSCs (such as IL1RN–IL1 receptor inhibitor, IL1RN–IL1 receptor) were related to anti-inflammatory responses^84^, and the loss of these pairs in between CECs and CSCs of KC was also observed (Fig. 6d). In the case of IL1RN–IL1 receptor, the ligand *IL1RN* expressed lower levels in KC ImCs, and the IL1 receptor (encoded by *IL1R1*) also showed a decreased expression in KC CSCs (Fig. 6e).

Furthermore, to access the potential role of cell–cell interactions in corneal stromal dysfunction of keratoconus patients, we performed NicheNet analysis^87^, which allowed us to predict cellular interactions by linking active ligand and target genes (Fig. 6f). Interestingly, NicheNet predicted that ImC-derived IL24 may induce the differential expression of *MMP1* in keratoconus CSCs. And *MMP2* expressed in CSCs was associated with ImC-derived *APOE* and *MMP9*. *MMP3* was associated with ImC-derived *MMP9* and CEC-derived *LGALS3*. Importantly, the metallopeptidase inhibitor gene *TIMP2* was associated with ImC-derived *APOE* and *POMC*. These results indicated that the balance of MMPs and TIMPs, a critical determinant of ECM integrity and function in corneal stroma, was potentially regulated by communications between CSCs and ImCs/CECs during keratoconus progression. Altogether, abnormal cell–cell communication patterns were shown to occur in keratoconus, which provided an explanation for keratoconus pathogenesis from a novel perspective.

## Discussion

**Fig. 7:**
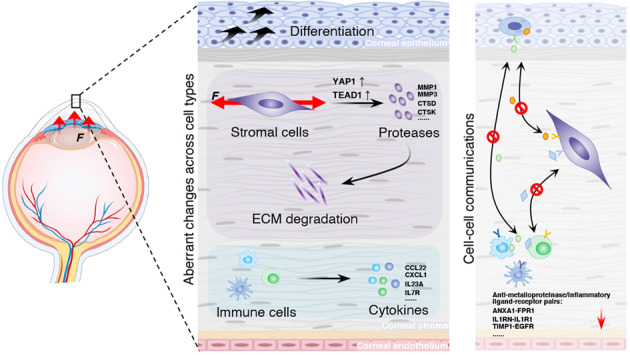
Model of keratoconus-related changes across cell types in the central cornea. In the cornea inflicted with keratoconus, mechanical stretch promotes the expression of several protease genes in stromal cells and aggravates ECM degradation, and the abnormal differentiation of corneal epithelial cells and elevated inflammatory signals were also detected (middle panel). Moreover, the dysregulated cell–cell communications occur in keratoconus corneas, especially for ligand-receptor pairs related to protease inhibiting and anti-inflammatory processes (right panel). The mechanical stretch exerted on cells was indicated by red arrows.

Keratoconus is a progressive ectatic corneal disorder that can lead to significant visual impairment, and usually starts during the teenage years. Corneal transplantation is the last resort in the treatment of keratoconus, placing perceived burdens on patients and society^1,2^. Despite the high prevalence of keratoconus, its pathophysiology is not completely clear. Thus, it is highly desirable to explore the molecular mechanisms underlying this intractable disease. Notably, a recent study by Collin et al. performed scRNA-Seq of central cornea samples obtained from two keratoconus patients and one healthy individual, revealing activation of collagenase in the corneal stroma and a reduced pool of limbal suprabasal cells^32^. Herein, we enlarged the sample numbers and systematically surveyed the cell type-specific transcriptional signatures between KC and Ctrl samples, and demonstrated the vital role of ECM degeneration in this disease, the implication of mechanical stretch in prompting protease production, and the corneal epithelial lesions (Fig. 7). The involvement of ImCs and dysregulated cell–cell communications in keratoconus were also characterized (Fig. 7), providing novel insights into the mechanisms related to keratoconus pathogenesis.

In this study, we identified the most predominant cell types in cornea, including corneal stromal and epithelial cells, with their heterogeneities deciphered. Besides, a small fraction of ImCs was arrested, comprising macrophage/monocytes, DC and T cells. In the classic view, stromal degeneration was generally considered as the cardinal symptom of keratoconus^10^. Then we defined the transcriptional signatures and DEGs from all cell types, confirmed the central role of CSCs in this disease, and implicated other cell types in keratoconus pathological processes. As mentioned, Collin et al. revealed the activation of collagenase in corneal stroma^32^. In our data, for CSCs, the up-regulated genes in keratoconus were involved in collagen metabolic process and ECM disassembly, consistent with the core characteristics of keratoconus^10^. Among these genes, two lysosomal cysteine proteases *CTSD* and *CTSK* were identified, providing potential biomarkers for keratoconus diagnosis. Here we also identified significantly increased oxidative phosphorylation and glycolysis in keratoconus, which expanded our understanding of the metabolic processes altered in keratoconus^59,60^. In addition, we revealed the cellular heterogeneity of corneal stroma and predicted keratoconus-associated subpopulations based on the expression specificity of risk genes. All these discoveries enhanced our knowledge of the molecular alterations in the corneas inflicted with keratoconus.

Eye rubbing, as a well-known risk factor for keratoconus development, can induce distinct alterations in corneal biomechanics^25,88^. However, little is known about the impact of stretch on corneal ECM degeneration. Previous studies reported that mechanical stretch could augment MMP expression in human fibroblasts isolated from keratoconus and healthy corneal stromal tissues^79,89,90^. Nevertheless, many fundamental questions remain poorly defined. How does mechanosensation impact gene transcription, and what is the contribution of mechanical stretch to ECM degeneration? Remarkably, we detected significantly elevated *YAP1* and *TEAD1* in keratoconus stromal cells. Further cyclical mechanical experiments on human corneal stromal cell lines demonstrated that mechanical stretch prompted the expression of several protease genes, including *MMP1*, *MMP3*, *CTSD* and *CTSK*, implying the activating effect of mechanical stretch on proteases during keratoconus progression. These results suggested that this risk factor not only changed corneal biomechanical characteristics, but also triggered considerable biochemical responses in CSCs. These findings coordinated with the pathogenic role of the biomechanics-enzymes axis in keratoconus development, providing novel insights into the pathogenesis of ectatic disorders. Further investigations were required to elucidate the in-depth mechanisms involved.

For corneal epithelium, abnormal differentiation signals were observed in keratoconus superficial cells. As reported in a recent study, the reduced pool of limbal suprabasal cells was observed in keratoconus^32^. Notably, in our study, we found that basal cells of the central cornea were decreased in keratoconus. Are there any associations between these two discoveries? And do highly differentiated superficial cells arouse the consumption of progenitor or basal cells? A recent study revealed that stretching induced skin expansion by creating a transient bias in the renewal activity of epidermal stem cells, and basal progenitors remains committed to differentiation^91^. In keratoconus corneal epithelium, does abnormal biomechanics induce the hyper-differentiation of basal cells? Further studies were needed to address these intriguing issues.

Beyond that, we also investigated alterations in ImCs. Keratoconus was traditionally regarded as a non-inflammatory disease, while accumulative evidence revealed the role of cytokines in keratoconus corneas and their microenvironment^92,93^. With the aid of single-cell data, it was not difficult to clarify this concern. In our study, we detected increased cytokines and cytokine-mediated signaling pathway in immune cells from keratoconus corneas, especially for DCs. Our data further demonstrated the contribution of inflammatory responses in keratoconus pathological processes at the single-cell resolution. Furthermore, accumulative evidence indicated that biomechanical stress was also closely related to inflammation modulation^94^, demonstrating that the biomechanical stress was probably involved in the inflammation responses of keratoconus, which needed to be further investigated.

In the survey of the variant cell–cell communications in KC and Ctrl corneas, numerous interactional pairs were identified between cell types, indicating that these cells participate in the maintenance of corneal homeostasis. However, a large number of ligand-receptor pairs related to multiple vital signaling pathways were erased in keratoconus. We spotted that ANXA1-FPR1, and IL1RN-IL1R1, whose anti-inflammatory roles have been reported, were erased in KC samples, implying their impact on the increased inflammation in keratoconus. In addition, TIMP1-EGFR, which was functioned as a metalloproteinase blocker in healthy corneas, were disappeared in keratoconus, indicating that its deficiency might be one of the underlying causes of keratoconus.

In this study, the KC samples were all procured from patients with severe keratoconus after DALK surgeries for subsequent studies. Due to the difficulty in obtaining the corneal specimens from patients with keratoconus in earlier stages^35^, it is challenging to determine the molecular mechanisms underlying the complete course of the disease. This potential limitation should be considered in this study. Although our results show that mechanical stretch prompted the expression of several protease genes, further experiments are needed to unravel the specific step-by-step mechanisms involved. Moreover, besides CSCs, the impacts of mechanical stretch on CECs and immune cells are interesting issues and are still remain to be discovered.

Collectively, our study delivered a comprehensive single-cell transcriptomic atlas for deciphering gene expression landscapes of heterogeneous cell types in KC and Ctrl corneas. These discoveries in keratoconus samples, including the dysregulated transcriptional signals in CSCs, the involvement of mechanical stretch in the production of ECM turnover enzymes, hyper-differentiated corneal superficial epithelium, and elevated cytokines in various immunocytes (Fig. 7), broaden our understanding of cell type-specific molecular alterations during keratoconus development. Moreover, we also observed that altered cell–cell communication may result in the disorder of normal cellular function (Fig. 7). Thus, these findings are potentially valuable for understanding the critical molecular mechanism underlying keratoconus and improving current preventative and therapeutic strategies for this disorder.

## Materials and methods

### Human samples

Human tissue collection was approved by the ethical committee of the Eye Hospital of Shandong First Medical University, and written informed consent was obtained before sample collection. The central corneal tissues of keratoconus patients were obtained during DALK surgeries that were performed at the Eye Hospital of Shandong First Medical University, and the specimens were composed of corneal epithelial and stromal layers. As healthy controls, post-mortem corneas were obtained from the eye bank of the Eye Hospital of Shandong First Medical University, and the corneal endothelium and limbus were removed. Briefly, for a fresh cornea tissue, the Descemet membrane-endothelial complex was peeled gently with surgical forceps under the stereoscope^95^, then the central cornea and limbus were separated with 8 mm trephine^96^. For all samples, central corneal tissues were recovered and preserved in Optisol-GS (Bausch & Lomb, Rochester, NY) for downstream experiments. See Supplementary Table S1 for detailed sample information.

### 10x Genomics scRNA-Seq

Single cells from each sample were independently processed into single-cell suspensions and library generations on 10× Genomics system. The cells were partitioned into GEM generation, barcoded cDNA library construction, and prepared using the single-cell 3’ mRNA kit (V2; 10× Genomics) as manufacturer’s directions. Then all libraries were subjected to quality tests (Fragment Analyzer 2100, Agilent Technologies) and sequencing (Platform: Illumina NovaSeq 6000; read length: 150 bp, paired-end).

### Data processing and downstream analysis

Transcripts were mapped to the corresponding reference genome (GRCh38-3.0.0 for human) using the10× Genomics CellRanger pipeline (version 3.1.0). The read count matrices for each sample were generated by CellRanger count. Then count data were imported into the Seurat R package (version 3.2.0)^77^, and quality control of each library was performed with the following steps: Cells with fewer than 500 genes detected or a mitochondrial gene ratio of greater than 10% were excluded, and genes expressed in fewer than 5 cells were removed; doublets were detected using the DoubletFinder package (version 2.0.2)^97^, the mean-variance-normalized bimodality coefficient (BCMVN) of each sample was calculated to determine the neighborhood size (pK_value), and the number of artificial doublets (pN_value) was set to 0.25; considering the dissociation-induced artifacts for sensitive cells, cells expressing a previously published dissociation-induced gene signatures were detected and removed gradually during the analysis if no other explainable marker genes were expressed^98^. After the above filtering pipeline, CCA method was used for libraries from different experimental batches to exclude batch effects in data integration^99^. Normalization was performed using LogNormalize method, and inherent variation caused by mitochondrial gene expression was regressed out. For cell clustering, principal component analysis (PCA) was performed on highly variable genes. The clustering at a resolution of 0.25 was performed for the top 13 PCs using the graph-based shared nearest neighbor method (SNN) (FindClusters function), and a total of 6 unsupervised cell clusters were obtained. Clustering results for individual or grouped samples were visualized using UMAP or t-SNE. Cell types were classified based on differential expression analysis, with cluster-specific marker genes identified (FindMarkers function).

### Identification of TFs using SCENIC

To identify active TFs in different cell types of the central cornea, we carried out a single-cell transcription factor network inference analysis using pySCENIC (version 0.10.3) as described^40^. Detailed descriptions of SCENIC is available on line at https://github.com/aertslab/SCENIC.

### Calculation of signature scores for metabolic process and DNA damage

For gene scoring analysis, gene sets were acquired from the MSigDB database, and the AddModuleScore function in Seurat R package was used to calculate the signature score of each gene set in each cell, and the two-sided Wilcoxon rank sum test was used for significance testing.

### Cell-cycle discrimination analysis

Assignment of cell cycle phase of individual cells was performed in Seurat using cell-cycle-specific expression data^100^. In brief, markers for G2/M and S phase were used for cell scoring, cells with neither G2/M nor S phase markers were regarded as being in G1 phase (CellCycleScoring function). Cells in each phase were quantified using the prop.table function.

### Subtype-specific enrichment of keratoconus-associated genes

With reference to the previous study^29^, for keratoconus-associated genes downloaded from OMIM Clinical Synopsis (https://www.omim.org), we used our single-cell transcriptomic data to relate the keratoconus association with patterns of subtype-specific expression. For each gene, we used one-sided Wilcoxon rank-sum test to assess its specificity within each subcluster, and used log-transformed *P* values as a measure of subtype specificity. *P* values smaller than 1e-300 were set to 1e-300 to avoid Inf log-scores, and the threshold of *P* value significance was 1e-5.

### Pseudotemperal trajectory analysis

We used the SCORPIUS package (version 1.0.7) to map cells onto pseudotime trajectories^66,67^. Analysis was performed on highly variable genes, and all other parameters were default. Individual CECs from each subcluster were subsequently placed onto linear pseudotime using the infer_trajectory function of the SCORPIUS package using default settings.

### Cell–cell communication analysis

We used CellPhoneDB (version 1.1.0, https://github.com/Teichlab/cellphonedb) to systematically predict cell-cell communications based on ligand–receptor analysis with default parameters^82^. Receptors or ligands expressed in at least 10% of cells of a given cell type and with a *P* < 0.05 were subsequently analyzed. For selected significant receptor–ligand pairs, we applied Circlize R package to visualize interaction links. Then we used Differential NicheNet (https://github.com/saeyslab/nichenetr), an extension to the default NicheNet algorithm^87^, to predict those active ligand–target links which were differentially expressed and contributed to keratoconus. CSCs was defined as “receiver/target” cell population in each niche. CECs and ImCs were defined as “sender/niche” cell population. Following calculating differential expression between the niches and ligand activities, prioritization of ligand-receptor and ligand-target links were accomplished. Top 50 ligands in keratoconus niche were selected and targets corresponding to these ligands with scores greater than 0.25 were retained. A quantile cutoff 0.33 was used on the ligand-target scores.

### Immunostaining experiments and antibodies

For immunohistochemical staining, the corneas were fixed in 4% paraformaldehyde, embedded in paraffin and then sectioned. After treatment with 3% H_2_O_2_, corneal sections were incubated with primary antibodies overnight at 4 °C and stained with an HRP-conjugated secondary antibody for 1 h at 37 °C (MaiXin Biotechnology, Fuzhou, China). All staining was examined under a Nikon fluorescence microscope. Antibodies used for immunohistochemical staining: rabbit anti-Cathepsin D (1:1000, Abcam ab75852), rabbit anti-Cathepsin K (1:1000, Abcam ab207086), rabbit anti-KRT80 (1:1000, Proteintech 16835-1-AP), rabbit anti-YAP1 (1:1000, Proteintech 13584-1-AP), rabbit anti-SPRR1B (1:1000, Proteintech 11959-1-AP).

For immunofluorescence staining, corneal tissues were fixed in 4% paraformaldehyde and blocked with 5% normal serum for 30 min at room temperature. The samples were treated with primary antibodies overnight at 4 °C and subsequently with secondary antibodies for 1 h at 37 °C. Nuclei were stained with 4',6 diamidino-2-phenylindole (DAPI). The flat mounts were examined and captured under a confocal microscopy (LSM800, Zeiss, Germany). Antibodies used for immunofluorescence staining: rabbit anti-K10 (1:1000, Abcam ab76318), rabbit anti-K12 (1:1000, Abcam ab185627), rabbit anti-PAX6 (1:1000, Proteintech 12323-1-AP).

### Cell culture and mechanical stretch application

The human stromal keratocyte cell line, HTK cells, were digested with 0.25% trypsin, and seeded at 6-well Bioflex plates (Flexcell Int. Corp., Hillsborough, NC, USA) with an initial density of 5 × 10^5^/well. After the cells reached a confluency of 80%, the cells were serum starved using FBS-free DMEM/F12 medium (Gibco) for 12 h. Then HTK cells were subjected to cyclical stretch (strain, 20%; frequency, 0.2 Hz) for 12 h using a Flexcell FX-5000 tension system (Flexcell Int. Corp, Hillsborrough, NC, USA). Cells plated on Bioflex plates but not subjected to stretch were used as control. The experiment was performed at 37 °C in a humidified incubator with 5% CO_2_. After the experiment, cells from each group were separately collected for further detections.

### RT-qPCR

Total RNAs were extracted using TransZol Up Plus RNA Kit (Transgen, Beijing, China), and 1 mg RNA was utilized as a template for reverse transcription with random hexamer primers using the cDNA Synthesis SuperMix Kit (TransGen, Beijing, China). RT-qPCR was conducted using SYBR qPCR Master Mix (Vazyme, Nanjing, China) on a Rotor-Gene Q system (Qiagen, Hilden, Germany). Each experiment was repeated three times independently. Relative gene expression data were analyzed using the comparative CT method (ΔΔCT). All RT-qPCR primer pairs are listed in Supplementary Table S7.

## Supplementary information

Supplementary information

Supplementary Table S2. DEGs for different cell clusters of human corneas

Supplementary Table S3. DEGs for different subclusters of CSCs

MATERIAL Supplementary Table S4. DEGs for different subtypes of CECs

Supplementary Table S5. Ligand-receptor pairs identified across cell types of KC samples

Supplementary Table S6. Ligand-receptor pairs identified across cell types of Ctrl samples

Supplementary Table S7. Information of primer sequences for RT-PCR

## Data Availability

All data needed to evaluate the conclusions in the paper are present in TEXT and the Supplementary Materials. All the raw and processed scRNA-seq files in this research have been deposited in the GSA database under accession code HRA000728. All other relevant data are within the paper and Supplementary files.
