# Publisher Correction: Single-cell atlas of keratoconus corneas revealed aberrant transcriptional signatures and implicated mechanical stretch as a trigger for keratoconus pathogenesis

**DOI:** 10.1038/s41421-022-00452-9

**Published:** 2022-08-12

**Authors:** Shengqian Dou, Qun Wang, Bin Zhang, Chao Wei, Huijin Wang, Ting Liu, Haoyun Duan, Hui Jiang, Mingna Liu, Xiaolin Qi, Qingjun Zhou, Lixin Xie, Weiyun Shi, Hua Gao

**Affiliations:** 1 Eye Institute of Shandong First Medical University, State Key Laboratory Cultivation Base, Shandong Provincial Key Laboratory of Ophthalmology, Qingdao, Shandong China; 2 Qingdao Eye Hospital of Shandong First Medical University, Qingdao, Shandong China; 3 Eye Hospital of Shandong First Medical University, Jinan, Shandong China; 4 School of Ophthalmology, Shandong First Medical University, Jinan, Shandong China

**Keywords:** Mechanisms of disease, Transcriptomics

Correction to: *Cell Discovery* (2022) 8:66

10.1038/s41421-022-00397-z published online 12 July 2022

During the production, Figs. 1 and 5 in the PDF version were misplaced. The Figs. 1 and 5 should have been appeared as below. We apologize for any inconvenience that it may have caused.

**Fig. 1:**
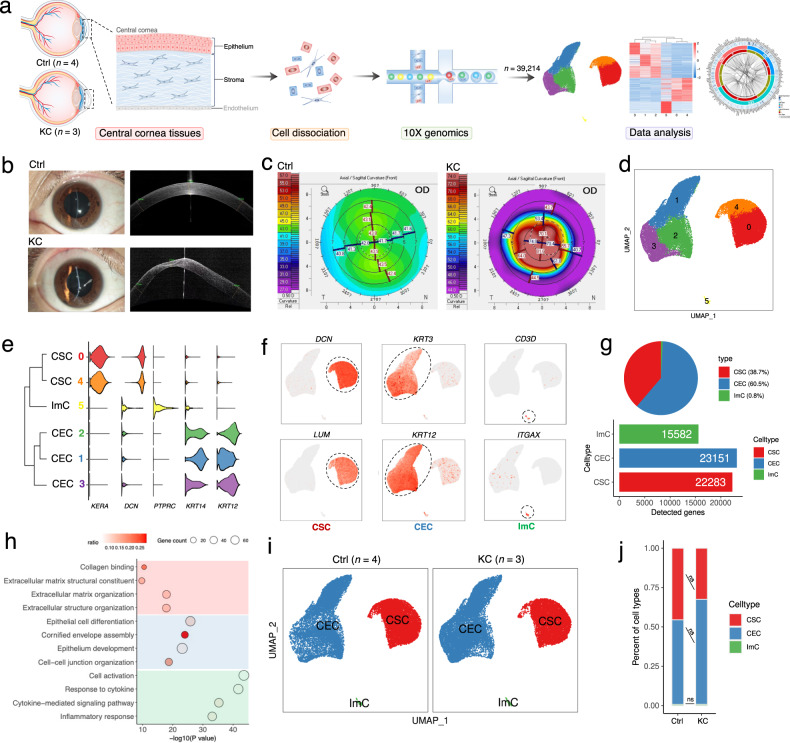
Overview of cellular compositions of KC and Ctrl human corneas delineated by scRNA-Seq analysis. **a** Overview of the experimental workflow in this study. In the schematic of diagram of the human cornea, the corneal epithelium and stroma (labeled in black) from the central cornea were subjected to downstream experiments, and the corneal endothelium (labeled in gray) was excluded (see Materials and Methods). **b**, **c** Anterior segment OCT (**b**) and Scheimpflug optical cross-sectional analysis (**c**) showed typical symptoms of keratoconus cornea. **d** UMAP representation of human corneal cells colored into 6 distinct clusters. **e**, **f** Expression levels (**e**) and distribution (**f**) of well-known representative cell markers across clusters. **g** The cell type proportions (top panel) and the number of detected genes per cell type (bottom panel). **h** Representative GO terms of specifically expressed genes in each cell type. **i** UMAP plot of human corneal cells colored by three major cell types in KC and Ctrl groups. **j** Bar plot representing the differences in relative proportion of major cell types between Ctrl and KC samples. ns, no significance (Student’s *t*-test). KC keratoconus; Ctrl control; CSC corneal stromal cell; CEC corneal epithelial cell; ImC immune cell.

**Fig. 5:**
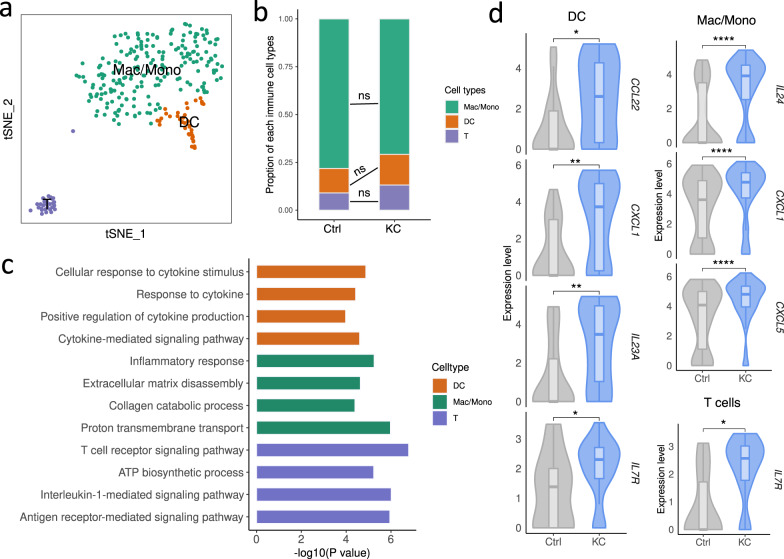
Inflammatory signals in keratoconus corneas contributed by immune cells. **a** t-SNE plot showing three immune cell types identified in KC and Ctrl samples. **b** Proportion of each immune cell type in KC and Ctrl samples. ns, no significance (Student’s *t*-test). **c** Representative GO terms of keratoconus-upregulated genes in each immune cell type. **d** Violin plots showing representative differentially expressed cytokines in keratoconus immune cells. *P < 0.05, **P < 0.001, ****P < 0.0001 (two-sided Wilcoxon rank-sum test).

The original article has been corrected.

