# Supplementary material for: Single-cell atlas of keratoconus corneas revealed aberrant transcriptional signatures and implicated mechanical stretch as a trigger for keratoconus pathogenesis
Source: Cell Discov. 2022 Jul 12;8:66. doi: 10.1038/s41421-022-00397-z (PMC9276680; doi:10.1038/s41421-022-00397-z)
Supplement: Supplementary file 1 — Supplementary information [file 41421_2022_397_MOESM1_ESM.pdf]

## Title

Single-cell atlas of keratoconus corneas revealed aberrant transcriptional signatures and implicated mechanical stretch as a trigger for keratoconus pathogenesis

## Running title

Single-cell transcriptomic atlas of keratoconus corneas

## Authors

Shengqian Dou[1,2], Qun Wang[1,2], Bin Zhang[1,2], Chao Wei[1,2], Huijin Wang[1,2], Ting Liu[1,2], Haoyun Duan[1,2], Hui Jiang[1,2], Mingna Liu[1,3,4], Xiaolin Qi[1,3,4], Qingjun Zhou[1,2], Lixin Xie[1,2], Weiyun Shi[1,3,4]*, Hua Gao[1,3,4]*

[1]Eye Institute of Shandong First Medical University, State Key Laboratory Cultivation Base, Shandong Provincial Key Laboratory of Ophthalmology, Qingdao, China.

[2]Qingdao Eye Hospital of Shandong First Medical University, Qingdao, China.

[3]Eye Hospital of Shandong First Medical University, Jinan, China.

[4]School of Ophthalmology, Shandong First Medical University, Jinan, China.

*Correspondence: Hua Gao (hgao@sdfmu.edu.cn) or Weiyun Shi (wyshi@sdfmu.edu.cn)

These authors contributed equally: Shengqian Dou, Qun Wang

**Supplementary figures and legends**

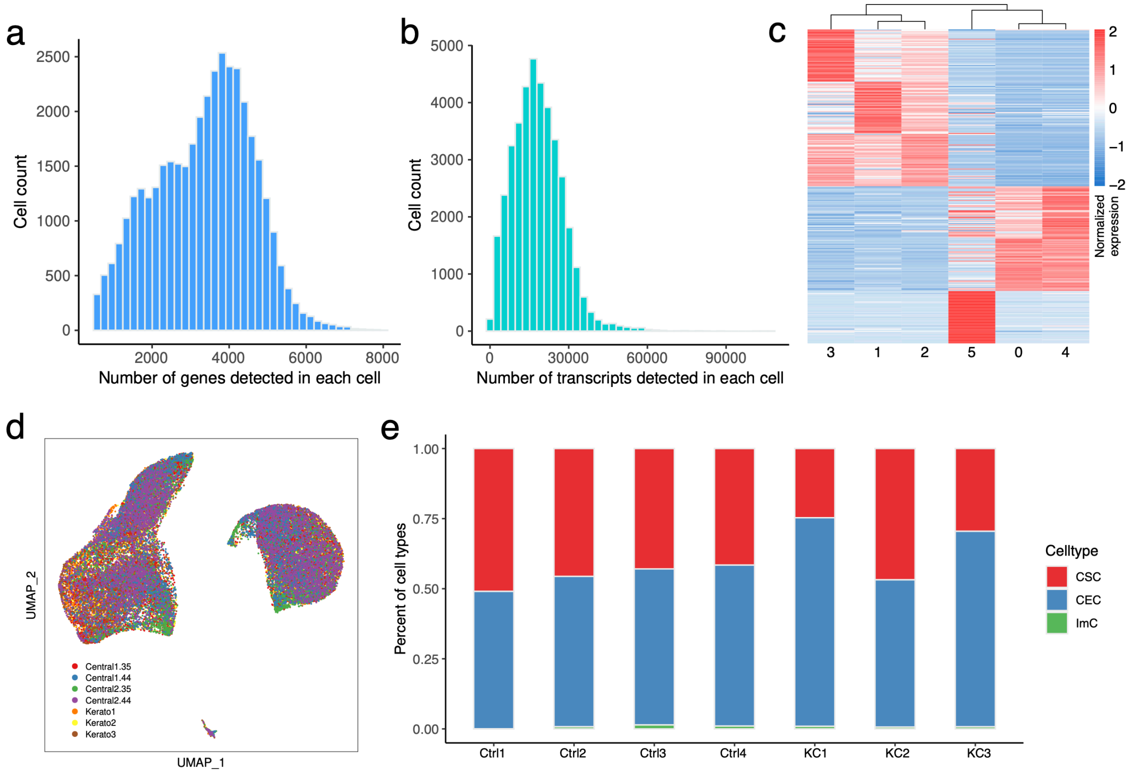


**Supplementary Fig. S1. Quality control and sampling information for each library.** a. Distribution of the cell number versus detected gene numbers. b. Distribution of the cell number versus the number of transcripts. c. Top 50 DEGs across cell clusters. The expression for each gene is row-scaled Z score. d. UMAP plot of human corneal cells colored by sample origins. e. Distribution of corneal cell types across samples.

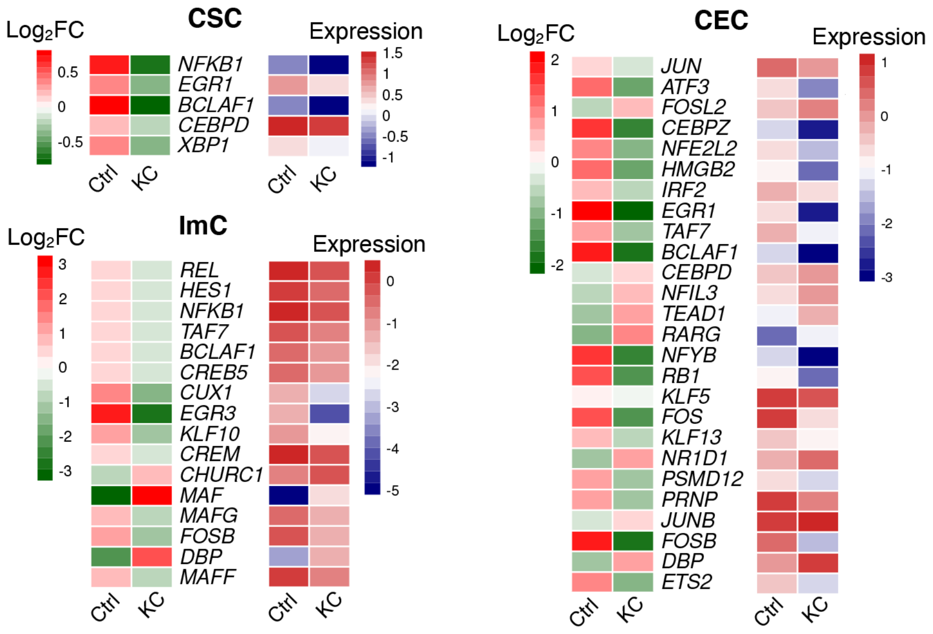


**Supplementary Fig. S2.** Heatmap showing $\log_2$ fold changes (left) and expression levels (right, with log2-transformed) of TFs from KC and Ctrl samples for each cell type.

a

| Gene | Fold Change | Pct.1 | Pct.2 | Pct.1/Pct.2 |
|---|---|---|---|---|
| *MMP1* | 1.79 | 0.845 | 0.804 | 1.05099502 |
| *MMP3* | 2.03 | 0.977 | 0.971 | 1.0061792 |
| *CTSD* | 1.88 | 0.876 | 0.763 | 1.14809961 |
| *CTSK* | 1.86 | 0.733 | 0.59 | 1.24237288 |

b

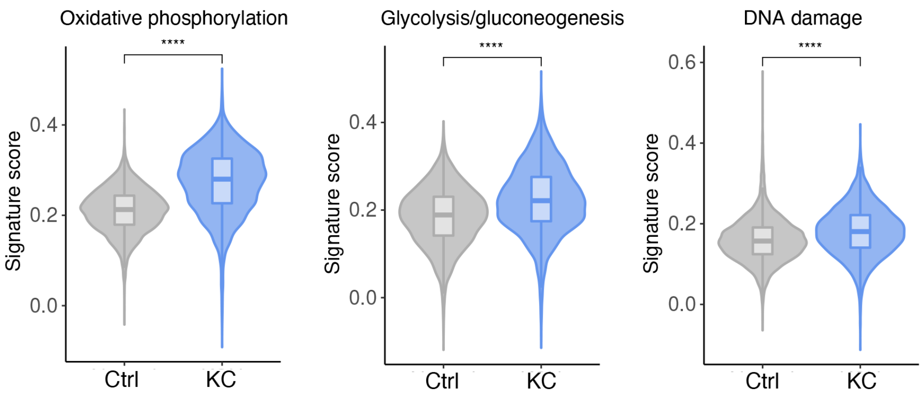


**Supplementary Fig. S3. Differential expression of representative genes and metabolic changes between KC and Ctrl corneas.** a. Expression changes of *MMP1*, *MMP3*, *CTSD* and *CTSK* in KC CSCs. Fold Change, the fold change of gene expression between KC and Ctrl samples; Pct.1, the percentage of cells expressing the given genes in KC samples; Pct.2, the percentage of cells expressing the given genes in Ctrl samples. Pct.1/ Pct.2, the ratio of percentage of cells expressing the given genes in KC and Ctrl samples. b. Changes of signature scores for metabolism and DNA damage in KC samples. ****$P < 0.0001$ (two-sided Wilcoxon rank-sum test).

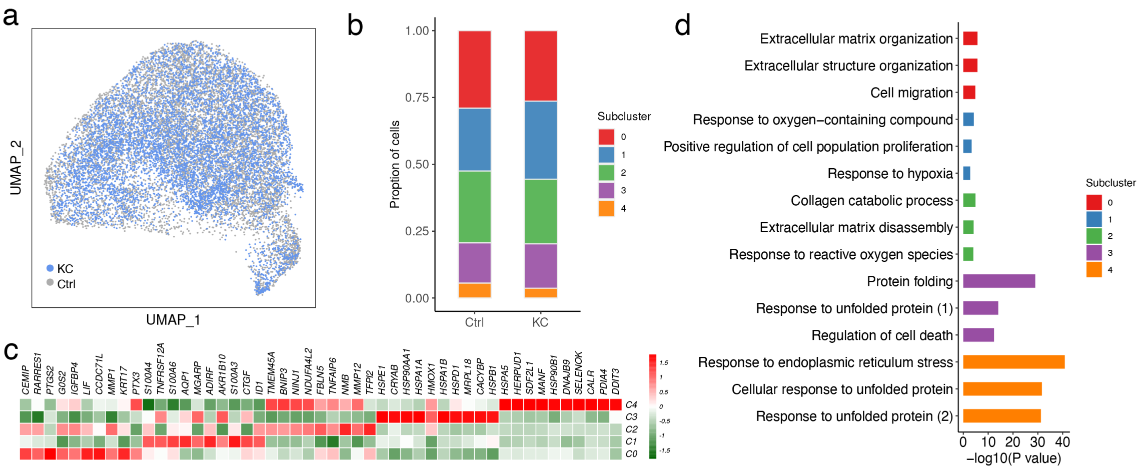


**Supplementary Fig. S4. Deciphering the heterogeneity of CSCs.** a. UMAP clustering of CSCs colored by KC and Ctrl groups. b. Distribution of five CSC subclusters across two groups. c. Expression patterns of top 10 differentially expressed genes for each cell subcluster. d. Representative GO terms of all differentially expressed genes in each subcluster.

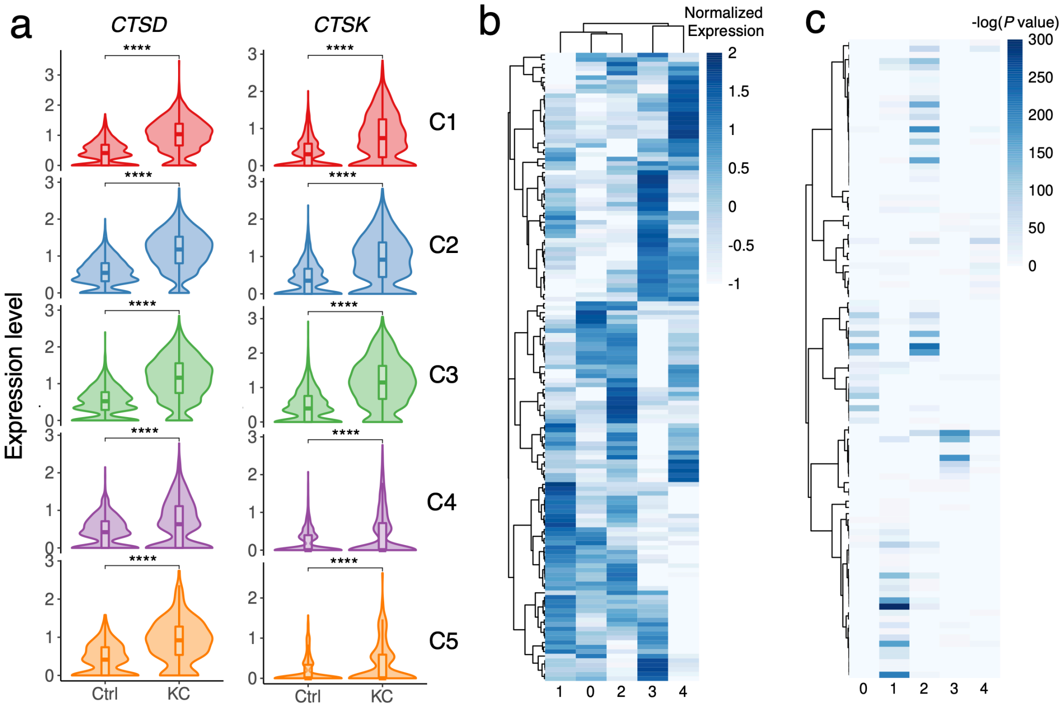


**Supplementary Fig. S5. Expression of keratoconus-related genes across cell subclusters.** a. Violin plots showing the differential expression of *CTSD* and *CTSK* between groups across subclusters. ****$P$ < 0.0001 (two-sided Wilcoxon rank-sum test) b. Relative expression of keratoconus-associated gene set across CSC subtypes. c. The enrichment of keratoconus-associated genes across corneal stromal subclusters. The enrichment scores are reported as the log-transformed $P$ values computed using one-sided Wilcoxon rank-sum test.

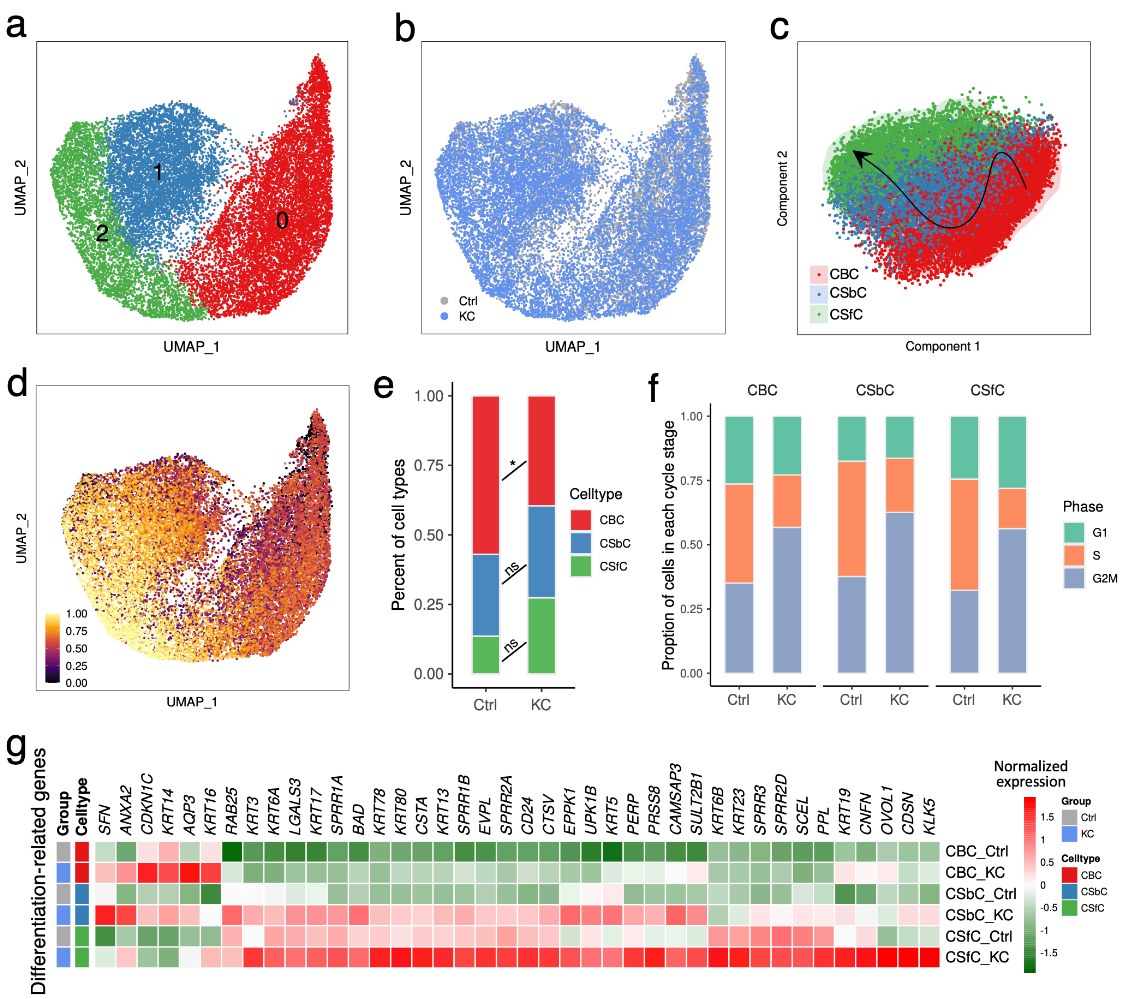


**Supplementary Fig. S6. Heterogeneous analysis of CEC subtypes.** a-b. UMAP visualization of CECs colored by subclusters (a) and groups(b). c. Inferred trajectory of corneal epithelial cells by SCORPIUS with cell types annotated. d. UMAP plot of corneal epithelial cells colored by pseudotime scores inferred by SCORPIUS. e. Bar graph representing the differences in relative proportion of corneal epithelial subtypes between Ctrl and KC samples. **P* < 0.05; ns, no significance (one-sided Wilcoxon rank-sum test). f. Proportion of cell cycle stages in both Ctrl and KC samples for each subtype. g. Expression of differentiation-related genes across CEC subtypes in Ctrl and KC samples.

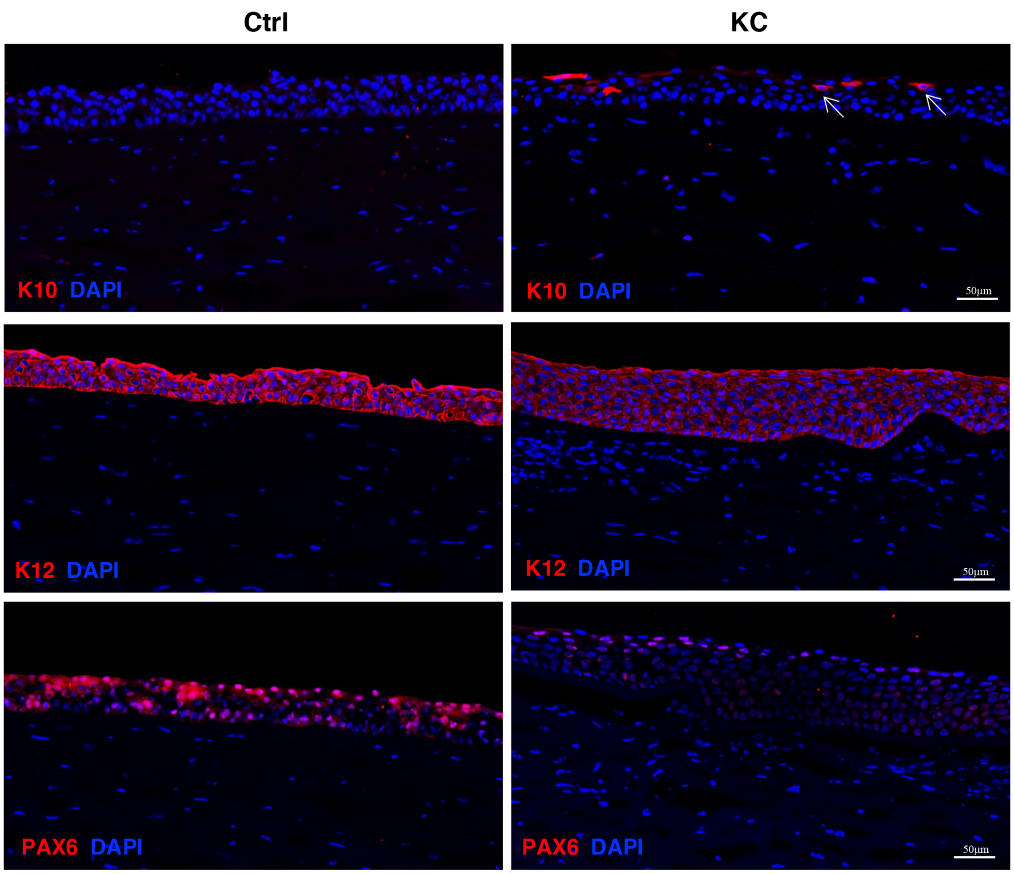


**Supplementary Fig. S7. Immunofluorescence analyses of K10, K12 and PAX6 in human corneal epithelium.**

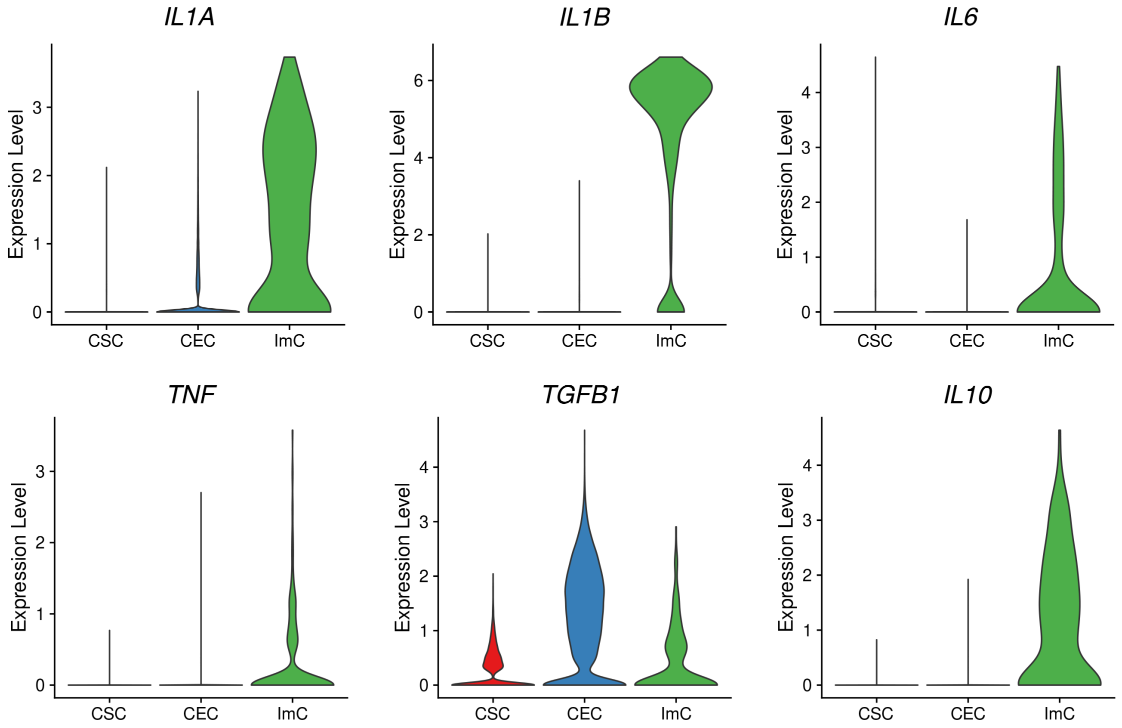

**Supplementary Fig. S8. Expression of several cytokines increased in keratoconus samples reported in previous studies.**

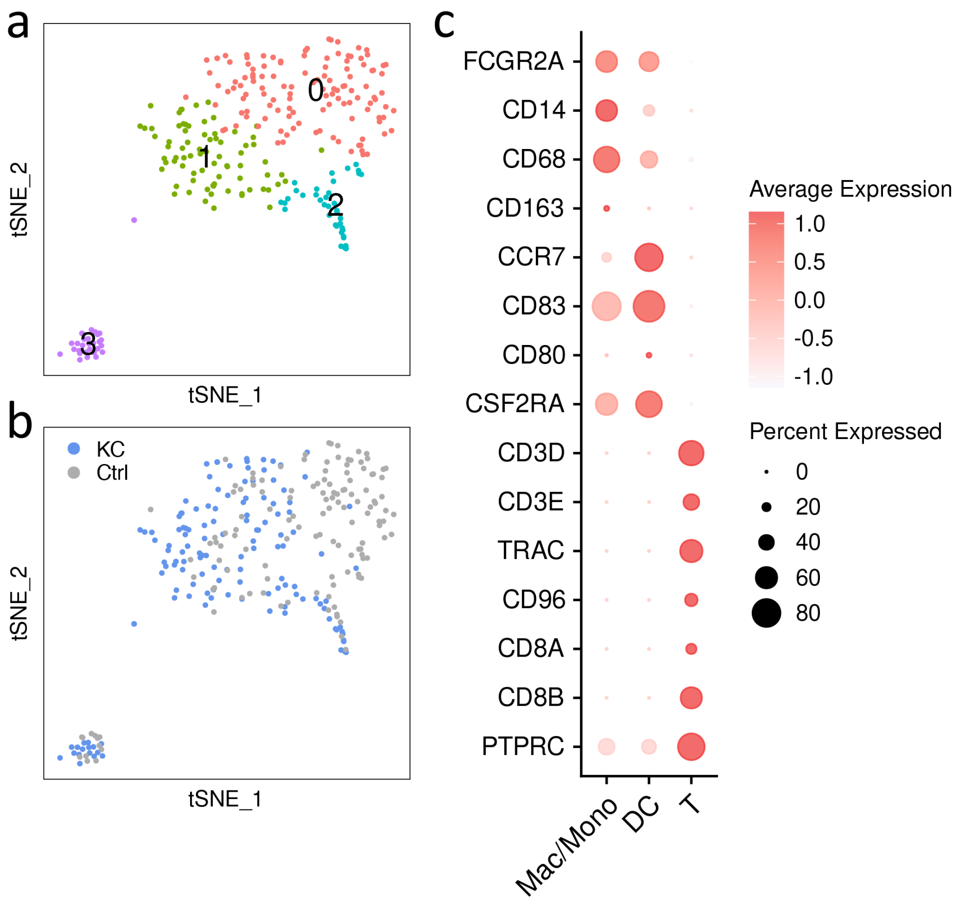


**Supplementary Fig. S9. Constitution of immune cells located in human central corneas.** a-b. t-SNE visualization of ImCs in central corneas colored by subcluster (a) and group (b). c. Expression of representative marker genes defining each type of ImCs.

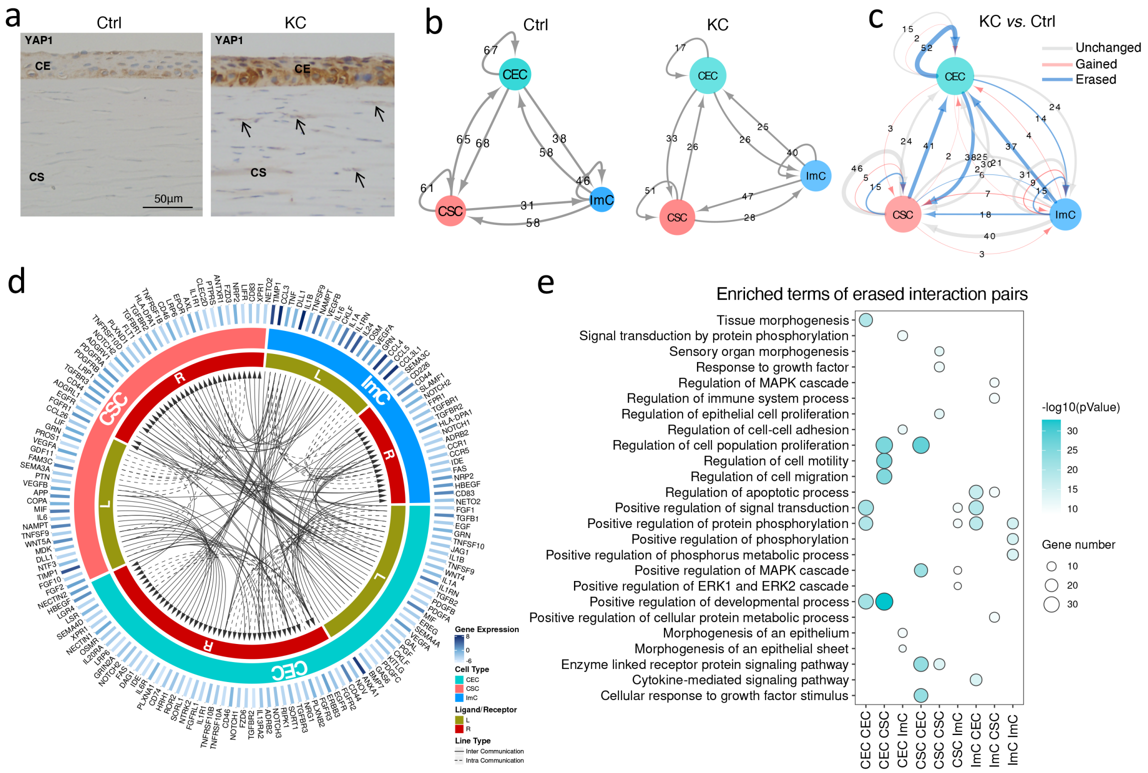


**Supplementary Fig. S10. Cell-cell interaction analysis.** a. Immunohistochemical analysis of YAP1 in corneal epithelium (CE) and corneal stroma (CS). b. Network visualization of ligand-receptor pair numbers among CSCs, CECs and ImCs in Ctrl (left) and KC (right) samples. c. The changed interaction events in KC compared to Ctrl samples. Cell-cell communications are indicated by the connected line. The line thickness is positively correlated with the number of ligand-receptor interaction events as annotated. d. Circos visualization of erased cell-cell interaction pairs in KC compared to that in Ctrl among three cell types. The gene expression values are calculated as the mean expression level of each gene in its corresponding cell type. Ligands and receptors were indicated by different colors. The intercellular communications are represented by solid lines, and the intracellular communications were represented by dashed lines. e. Representative GO enrichment terms for erased pairs in KC samples compared to those in Ctrl.

**Supplementary Tables**

Supplementary Table S1. Information for samples included in this study.

| Donor ID | Donor 1 | Donor 1 | Donor 2 | Donor 2 | Donor 3 | Donor 4 | Donor 5 |
|---|---|---|---|---|---|---|---|
| Sample ID | Ctrl1 | Ctrl2 | Ctrl3 | Ctrl4 | KC1 | KC2 | KC3 |
| Age | 35 | 35 | 44 | 44 | 25 | 19 | 19 |
| Gender | Male | Male | Male | Male | Male | Male | Male |
| Time to preservation | 6h | 6h | 1.5h | 1.5h | 1h | 1h | 1h |
| Disease | Ctrl | Ctrl | Ctrl | Ctrl | KC | KC | KC |
| Stage | / | / | / | / | Severe | Severe | Severe |
| Filtered cells | 5,497 | 4,262 | 4,129 | 5,014 | 6,915 | 5,369 | 8,028 |
| nGene | 21,750 | 22,538 | 19,268 | 20,363 | 18,716 | 19,330 | 19,860 |

Supplementary Table S2. DEGs for different cell clusters of human corneas (xls).
Supplementary Table S3. DEGs for different subclusters of CSCs (xls).
Supplementary Table S4. DEGs for different subtypes of CECs (xls).
Supplementary Table S5. Ligand-receptor pairs identified across cell types of KC samples (xls).
Supplementary Table S6. Ligand-receptor pairs identified across cell types of Ctrl samples (xls).
Supplementary Table S7. Information of primer sequences for RT-PCR (xls).
